# Genetic Mapping of the Root Mycobiota in Rice and its Role in Drought Tolerance

**DOI:** 10.1186/s12284-023-00641-4

**Published:** 2023-05-22

**Authors:** Beatriz Andreo-Jimenez, Dennis E. te Beest, Willem Kruijer, Nathan Vannier, Niteen N. Kadam, Giovanni Melandri, S. V. Krishna Jagadish, Gerard van der Linden, Carolien Ruyter-Spira, Philippe Vandenkoornhuyse, Harro J. Bouwmeester

**Affiliations:** 1 Laboratory of Plant Physiology, Wageningen University and Research, Wageningen, The Netherlands; 2 Biointeractions and Plant Health, Wageningen University and Research, Droevendaalsesteeg 1, 6708PB Wageningen, The Netherlands; 3 Biometris, Wageningen University and Research, Wageningen, The Netherlands; 4 EcoBio, CNRS, Université de Rennes I, Rennes, France; 5 International Rice Research Institute, Los Baños, Laguna, Philippines; 6 Centre for Crop Systems Analysis, Wageningen University and Research, Wageningen, The Netherlands; 7 Kansas State University, Manhattan, KS 66506 USA; 8 Plant Breeding, Wageningen University and Research, Wageningen, Netherlands; 9 School of Plant Sciences, University of Arizona, Tucson, USA; 10 Plant Hormone Biology Group, Swammerdam Institute for Life Sciences, University of Amsterdam, Science Park 904, 1098 XH Amsterdam, The Netherlands

**Keywords:** Drought, Fungi, Root mycobiota, GWAS, *Oryza sativa* (rice), Roots

## Abstract

Rice is the second most produced crop worldwide, but is highly susceptible to drought. Micro-organisms can potentially alleviate the effects of drought. The aim of the present study was to unravel the genetic factors involved in the rice-microbe interaction, and whether genetics play a role in rice drought tolerance. For this purpose, the composition of the root mycobiota was characterized in 296 rice accessions (*Oryza sativa* L. subsp. *indica*) under control and drought conditions. Genome wide association mapping (GWAS) resulted in the identification of ten significant (LOD > 4) single nucleotide polymorphisms (SNPs) associated with six root-associated fungi: *Ceratosphaeria* spp., *Cladosporium* spp., *Boudiera* spp., *Chaetomium* spp., and with a few fungi from the Rhizophydiales order. Four SNPs associated with fungi-mediated drought tolerance were also found. Genes located around those SNPs, such as a DEFENSIN-LIKE (DEFL) protein, EXOCYST TETHERING COMPLEX (EXO70), RAPID ALKALINIZATION FACTOR-LIKE (RALFL) protein, peroxidase and xylosyltransferase, have been shown to be involved in pathogen defense, abiotic stress responses and cell wall remodeling processes. Our study shows that rice genetics affects the recruitment of fungi, and that some fungi affect yield under drought. We identified candidate target genes for breeding to improve rice-fungal interactions and hence drought tolerance.

**Supplementary Information:**

The online version contains supplementary material available at 10.1186/s12284-023-00641-4.

## Introduction

Rice (*Oryza sativa* L.) is the most important staple food crop worldwide, with an average production of around 700 million tons per year (FAOSTAT database: http://www.fao.org/faostat/en/#home). Rice is a high yield crop when grown in paddy fields under waterlogged conditions. Waterlogged conditions guarantee high rice productivity while preventing the growth of weeds, but are also responsible for the low water use efficiency (0.60 kg yield/m^3^ water) of rice compared with other major cereals, such as maize (2.19 kg yield/m^3^ water) and wheat (2.46 kg yield/m^3^ water) (Liu et al. 2009; Kuschel-Otárola et al. 2020). Global climate change has increased both the intensity and duration of drought periods (Trenberth et al. 2014) thereby reducing the availability of water for irrigation and threatening the production of high water-demanding crops including rice grown in paddy fields.

Plants have several physiological and developmental responses to mitigate the effects of drought, including adjusting flower phenology, closing the stomata to reduce transpiration, developing a deeper root system to increase water uptake, accumulating stress-protective proteins and metabolites (Cattivelli 2008; Pandey & Shukla 2015; Melandri et al. 2020a, b) and establishing mutualistic symbioses with micro-organisms, collectively called the microbiome (Vandenkoornhuyse et al. 2015; Santos-Medellín et al. 2017; Xu et al. 2018; Andreo-Jimenez et al. 2019; Simmons et al. 2020; Abedini et al. 2021). Plant microbiomes are present in/on all plant organs including roots and shoots, and are composed of diverse bacteria and fungi (Sessitsch et al. 2011; Hardoim et al. 2015). Root-associated fungi are important components of the plant microbiome because of their positive effect on plant tolerance of both biotic stresses (Mejía et al. 2008; Maciá-Vicente et al. 2008; Chadha et al. 2015) and abiotic stresses, including drought (Redman et al. 2011; Worchel et al. 2012; Azad & Kaminskyj 2015; Santos-Medellín et al. 2017). Root-associated fungi are ubiquitous, they colonize the roots of all land plants. Arbuscular mycorrhizal (AM) fungi are a particular case of root-associated fungi, although not very abundant in rice (Yuan et al. 2010; Andreo-Jimenez et al. 2019), they have been shown to play an important role in drought tolerance by improving plant photosynthetic efficiency and boosting the accumulation of antioxidants under stress (Ruiz-Sánchez et al. 2010; Duc et al. 2018). On the other hand, these symbionts can also increase susceptibility to pests and pathogens e.g. *Lissorhoptrus oryzophilus*, *Rhizoctonia solani* (Bernaola et al. 2018). Other non-mycorrhizal fungal endophytes, such as *Fusarium* sp. (Hypocreales), *Curvularia* sp. (Pleosporales) and *Cladosporium* sp. (Capnodiales), have also been shown to enhance drought tolerance in plants. *Nicotiana benthamiana* and rice inoculated with fungal endophytes produced more biomass and grain, higher water content and/or increased activity of antioxidant enzymes than non-inoculated plants under drought or salinity stress (Redman et al. 2011; Dastogeer et al. 2018). In contrast to AM fungi, non-mycorrhizal fungal endophytes do not have a tight relationship with their host and their effect on plants depends more on the host and environmental conditions (Rodriguez et al. 2008; Geisen et al. 2021). Strigolactones have been shown to play an important role in recruitment and colonization by AM fungi of rice growing under phosphorus deficiency (Choi et al. 2020; Kim et al. 2022). However, our understanding of the mechanisms that underlie the recruitment of AM and other fungi by rice under drought and the recruitment dependency on the environment is far from complete.

The host plant genotype has been shown to influence the structure and composition of the microbial community in leaves (Horton et al. 2014; Wagner et al. 2016; Leopold & Busby 2020) and roots (Edwards et al. 2015; Liu et al. 2019; Brown et al. 2020) but little is known about the mechanisms behind this relationship. Plant–microbe associations are complex, and studies using holistic approaches that account for both environmental and genetic variables as drivers of these relationships are required to advance our understanding of these systems (Salvioli & Bonfante 2013).

Like plant response to drought and yield, microbiome assembly in plants is a complex trait that is challenging to address using reverse genetics approaches. With the development of faster and cheaper sequencing technologies, forward genetic strategies such as whole genome studies have become an important tool to study such complex traits (Han & Huang 2013; Zargar et al. 2015). Genome wide association studies (GWAS) use genetic maps made of thousands of single nucleotide polymorphism (SNP) markers that allow the genetic dissection and fine mapping of complex traits (Brachi et al. 2011; Beilsmith et al. 2019). Thanks to new generation high throughput sequencing techniques, dense genetic marker maps have become available for several crops, including rice (Zhao et al. 2011; McCouch et al. 2016). In the last decade, several GWAS have been performed of rice traits including grain size, starch quality, aluminum and iron toxicity tolerance, panicle morphology, canopy temperature, root architecture and grain yield (Huang et al. 2010; Famoso et al. 2011; Rebolledo et al. 2016; Kadam et al. 2017; Melandri et al. 2020b, 2021). The use of GWAS to map variations in plant traits that are related to the interaction with microorganisms is a novel research area. A pioneer study using the model plant *Arabidopsis thaliana* identified loci associated with bacterial and fungal endophyte community abundance in the phyllosphere (Horton et al. 2014). These loci were mainly involved in processes linked to the cell wall, such as xyloglucan biosynthesis and trichome development. A GWAS on root associated bacteria and fungi in *Arabidopsis* identified loci encompassing genes related to plant defense but also to root development (Bergelson et al. 2019). Furthermore, the same authors concluded that fungi strongly influence the root microbiome, and that the host effect on the fungal microbiome is stronger than on the bacterial community. So far, GWA mapping of the microbial community in crops has only been used for bacteria on maize and rice leaves (Wallace et al. 2018; Roman-Reyna et al. 2020), and sorghum roots (Deng et al. 2021). Recently, QTL mapping showed that host genetics shapes the bacterial and fungal communities in the rhizosphere of barley and tomato (Escudero-Martinez et al. 2022; Oyserman et al. 2022), however, how this is affected by environmental constraints such as drought remains to be explored.

The primary objective of the present study was to understand how host genetics affects the association between rice and root-associated fungi and how these fungi contribute to drought tolerance in rice. To achieve this objective, we investigated (1) the role of the rice as host in its association with root-associated fungi, (2) the changes in this partnership under drought conditions, (3) the extent to which the fungal community affects yield in rice under well-watered compared with under drought conditions, and (4) the genetic loci associated with yield and the recruitment of root-associated fungi.

## Materials and Methods

### Plant Material and Field Experiment

For this study, 296 accessions of *Oryza sativa* (L.) subsp. *indica* were used in a field experiment (14°11′ N, 121°15′ E; elevation 21 m above sea level) conducted at the International Rice Research Institute (IRRI) in the 2013 dry season. The panel was representative of the genetic diversity of *indica* rice from tropical and sub-tropical regions and includes both traditional landraces and improved lines (Rebolledo et al. 2016). The 25 × 90 m experimental field was located on an upland farm in a loamy soil with a mix of clay (36%), sand (22%) and silt (41%). Plants were sown in 2.5 × 0.8 m plots, each containing 48 plants (four rows of 12 plants) of one accession. To reduce the effect of field variation, the plots were laid out in a serpentine design with a total of three replicates per accession and per treatment. The field was split into two parts, one well-watered and the other under drought conditions. To synchronize flowering, the accessions were divided into groups according to days to flowering (data collected previous to this study, Kadam et al. 2018), and progressively sown and transplanted with intervals of 10 days between each group. All the plants were grown in flooded conditions until they reached the 50% flowering stage (i.e. when half the plants in each plot were in flower). At this point, in half of the field, watering was withheld for 14 days (soil water potential − 46 kPa) (drought treatment) while the other half was kept flooded (well-watered treatment). At the end of the drought treatment, the stress field was re-watered until all the accessions reached maturity for harvest when grain yield was determined (for details, see Kadam et al. 2018). At the end of the 14 day period of stress, root samples were collected by taking 10 × 70 cm soil cores from the inner part of each plot. Samples were placed in plastic bags and stored at 4 °C until further processing. The 1776 root samples were placed on a fine sieve and carefully cleaned under a gentle stream of tap water then stored at − 80 °C until further analysis. This cleaning process was chosen to focus on the root associated fungi (i.e. both root-epiphytic and -endospheric fungi).

### DNA Extraction and Sequencing

Roots from the same accession and treatment (biological replicates) were pooled and ground to a powder with a mortar and pestle in liquid nitrogen. DNA was extracted from 60–80 mg of plant material using the DNeasy 96 Plant Kit (Qiagen) following the manufacturer’s protocol. The DNA was diluted tenfold for amplification of a fragment of the 18S SSU rRNA gene using general fungal primers (NS22: 5′-AATTAAGCAGACAAATCACT-3′ and SSU0817: 5′-TTAGCATGGAATAATRRAATAGGA-3′) (Borneman & Hartin 2000), with the following program: 95 °C for 4 min; 40 cycles at 95 °C for 30 s, 54 °C for 30 s and 72 °C for 1 min; and a final extension at 72 °C for 7 min. Primers were modified to allow amplicon multiplexing for the sequence production process: a collection of 96 modified SSU0817 primers was generated each containing a specific tag consisting of 10 nucleotides. The PCR products were purified with AMPure XP beads (Beckman Coulter) using a genomic sample preparation platform (BRAVO, Agilent technologies). The concentration of amplicons was measured with an ultrasensitive fluorescent stain (Quant-ITTMPicoGreen^®^dsDNA Assay kit, Invitrogen), and the size was determined using the Agilent High Sensitivity DNA kit (Agilent Technologies). Equimolar amplicon pools were paired-end sequenced (Illumina Miseq), following the manufacturer’s guidelines. The raw data were uploaded in the NCBI database under project number PRJNA810017.

### Trimming and Identification of Sequences

The 5’ and 3’ primers (on R1 and R2 reads) and unknown bases (n) were removed from the sequences using the Phyton package ‘Cutadapt’ (Martin 2011). The reads were analyzed according to FROGS pipeline guidelines (Kozich et al. 2013; Escudié et al. 2018) using the galaxy workbench at Genotoul (http://bioinfo.genotoul.fr). The clustering step was performed with the ‘swarm’ method thus avoiding the need for an identity threshold. The sequence-clusters (Operational Taxonomic Units, OTUs) produced by FROGS use a similar view as the amplicon sequence variant (ASV) delineation (Callahan et al. 2017) but with the advantage of limiting the risk of overestimating diversity if copies of the SSU rRNA gene differ within a given individual. Following the pipeline designer’s advice, a de-noising step was performed with a maximum distance of aggregation of 1 followed by a second step with a maximum distance of aggregation of 3. Chimera products were filtered out with the FROGS ‘remove chimera’ tool. A filter was then applied to remove sequence-clusters with sequences in less than five samples to avoid artificial sequence clusters. Sequence clusters were identified with ‘Blast + ’ (Camacho et al. 2009) using the Silva 128 18S database. Sequence cluster assignments were filtered based on the quality of the match with a Blast threshold of 95% and 95% Blast identity. Subsequently, all sequence clusters were checked at the phylum level and those belonging to a non-fungal taxon were removed.

### Microbial Ecology Analyses

All statistical analyses were performed in R (R core team 2021). The contingency matrix of the sequence cluster count was filtered: sequence-clusters that occurred at least 25 times with a total of at least 100 reads were retained. Drought and control samples were filtered separately. For each sample, the counts assigned to each sequence cluster were log-transformed, i.e. divided by its total, summed with a pseudo-fraction (0.001), and then log transformed. The thus log-transformed data were adjusted for the plate effect (using the control and drought samples together), using a linear model, *removeBatchEffect*, in the R package LIMMA (Ritchie et al. 2015). This normalized contingency matrix was used for the follow up analyses. Data on rice grain yield were averaged across replicates. From the normalized contingency matrix, sequence cluster richness, evenness and diversity index estimators were calculated using the VEGAN R package (Oksanen et al. 2015) and BIODIVERSITY R (Kindt & Coe 2005). Statistical differences in these parameters were analyzed using two-way ANOVA with the R package CAR (Fox & Weisberg 2011). In order to reduce the size of the sequence-cluster dataset and to extract the most relevant information concerning the sequence-cluster effect on yield, the differences within the fungal community under the different treatments were analyzed using redundancy analysis (RDA) with grain yield as constraining covariate, using the function *rda* in the VEGAN R package. Redundancy analysis summarizes the variation in the microbial community that is explained by yield and drought. To test for significant differences between fungal communities under control and drought treatments, a permutational multivariate analysis of variance (PERMANOVA) was run with the *adonis* function on the Bray–Curtis distance matrix from the relative abundances using the VEGAN R package.

### Population Genotyping

We worked with a collection of 296 *indica* accessions, of which 274 were included in a bigger panel that was genotyped (genotyping-by-sequencing) with ~ 90,000 SNPs at Cornell University, USA (Rebolledo et al. 2016; Kadam et al. 2017). These 274 accessions were used for association mapping analysis.

### Genome-Wide Association Analysis

Marker-based estimates of narrow-sense heritability were computed based on all the cultivars used in the present study (Kruijer et al. 2015). GWA analyses were performed of all sequence clusters with a frequency higher than 5%, using the 82,858 SNPs with a minor allele frequency (MAF) > 0.05. Univariate GWAS was performed of each fungal sequence cluster under drought and control conditions separately, as well as for the RDA axes described above. We used the approach of Kang and colleagues (Kang et al., 2010) implemented in the STATGENGWAS package (https://cran.r-project.org/web/packages/statgenGWAS/index.html) using the function *runSingleTraitGwas*. For the sequence-cluster univariate GWAS, we only used the most abundant fungal taxa (17 sequence clusters under control and 15 under drought conditions). For the remaining analyses, we selected the sequence clusters that occurred at least 150 times with a total number of reads of at least 5000. All SNPs with an FDR < 0.10 were selected.

### Linkage Disequilibrium Analysis and Identification of Candidate Genes

The linkage disequilibrium (LD) decay rate in *indica* rice varieties is about 75 Kb (Mather et al. 2007). We consequently defined windows of 75 Kb upstream and 75 Kb downstream of the SNP of interest to search for candidate genes using the TRIO R package (Schwender et al. 2014). Genes located in the selected windows were obtained from the MSU Rice Genome Annotation Project database (http://rice.plantbiology.msu.edu), version 7.0. For 24 loci, in silico gene expression analyses were performed with RiceXPro (http://ricexpro.dna.affrc.go.jp/) and IC4R Rice Expression Databases (http://expression.ic4r.org/).

## Results

### Rice Genetic markers Associated with Root Mycobiota

To study the role of the host in the recruitment of root mycobiota and how the recruitment changes under drought, a root fungal community was analyzed to identify which sequence clusters differed the most between well-watered and drought conditions. Univariate GWA analysis was used to search for genomic associations with the most abundant sequence clusters.

The root fungal community sequencing data resulted in a total of 15,271,794 sequences belonging to 2,687 different sequence clusters. A total of 2,347 sequence clusters were found in both treatments, while 61 were specific to the drought treatment and 278 to the control treatment (Additional file 1: Fig. S1a). The total number of sequence reads (abundance) and the total number of sequence clusters (richness) were lower under drought conditions, while the diversity (Shannon) and species balance (J-evenness) indexes were higher under the drought treatment (Additional file 1: Fig. S1b). The fungal community observed in our experiment consisted of 12 different phyla, the most abundant group being Ascomycota, followed by Basidiomycota. Glomeromycota, the group of fungi that form arbuscular mycorrhiza, were underrepresented with only four sequence clusters (Additional file 1). Although Glomeromycota richness may have been underestimated as a result of primer bias during amplification, this bias would be expected to be similar across samples. Under drought there was an increase in the relative abundance of sequence clusters belonging to Dothideomycetes (Pleosporales: 1.7-fold increase, Capnodiales: 1.5-fold increase), followed by Eurotiomycetes and Chytridiomycetes (Additional file 1: Fig. S1a) while under control conditions, the most abundant sequence clusters belonged to Sordariomycetes (Sordariales: twofold higher, Pezizales: 1.6-fold higher). The few Glomeromycota sequence clusters detected were two fold more abundant in the control treatment (Additional file 2). The drought treatment resulted in a significantly different fungal microbiome from the microbiome found under control conditions (Additional file 1: Fig. S2) (PERMANOVA analysis control *vs.* drought; R^2^ = 0.70; *P* = 0.001). As treatment had a strong effect on the composition of the fungal community, the two treatments were analyzed separately from then on.

**Fig. 1:**
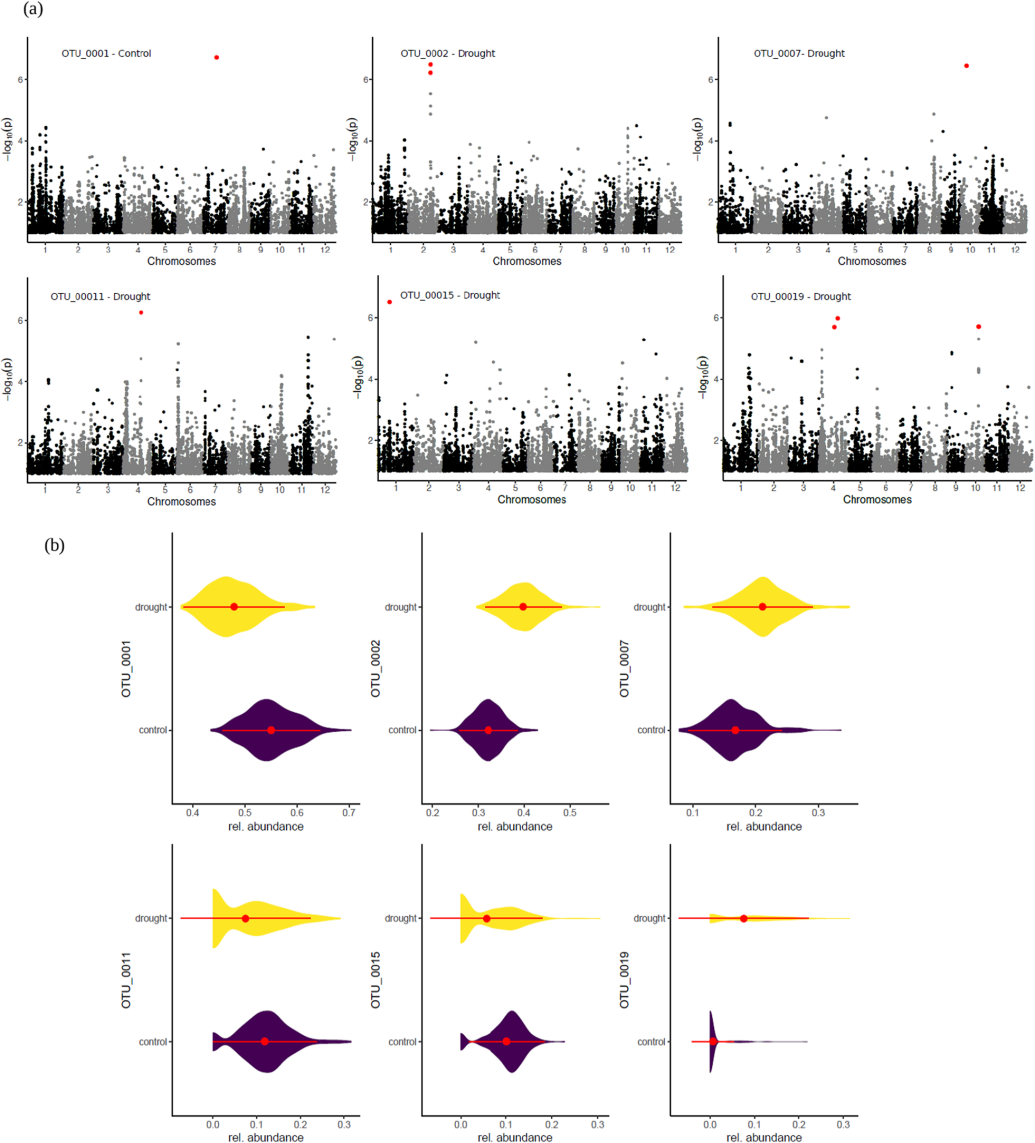
Host effect on fungal associations. **a** Manhattan plots showing SNPs linked to the abundance of six fungal sequence clusters present in the microbial community. **b** Violin plots showing the relative abundance of the sequence clusters under the control and drought treatments

Univariate GWA analysis was used to identify the loci associated with the abundance of each individual sequence cluster. Analysis of marker-based narrow-sense heritability showed that 7% of the sequence-clusters had heritability (h^2^) values ranging from 0.5 to 0.2, 12% from 0.2 to 0.1 and the rest below 0.1. The analysis yielded ten SNPs that were significantly associated with six different sequence clusters (FDR < 0.10) (Fig. 1a; Additional file 2), the majority of which were found under drought conditions, thereby confirming that fungal associations with rice roots are affected by rice genetics, and that the effect differs under drought and control conditions. The significant SNPs were located on chromosomes 7 (control treatment), and chromosomes 1, 2, 4 and 10 (drought treatment), and the SNPs had an allelic effect with A/G, C/G and C/A changes (Fig. 1a; Additional file 1: Fig. S3). Furthermore, the relative abundance of sequence clusters with a significantly associated SNP was found to differ between treatments, with half the sequence clusters being more abundant and the other half being less abundant in the drought treatment than in the control treatment (Wilcoxon, *P* < 0.001) (Fig. 1b). Among sequence clusters with a significantly associated SNP, two belong to the order Rhizophydiales, one to the Chaetomiaceae, and the rest are a *Cladosporium* sp., *Ceratosphaeria* sp. and *Boudiera* sp. (Additional file 1: Table S1).

### Rice Genetic Markers Related to Root Associated Fungi that Affect Grain Yield

**Fig. 2:**
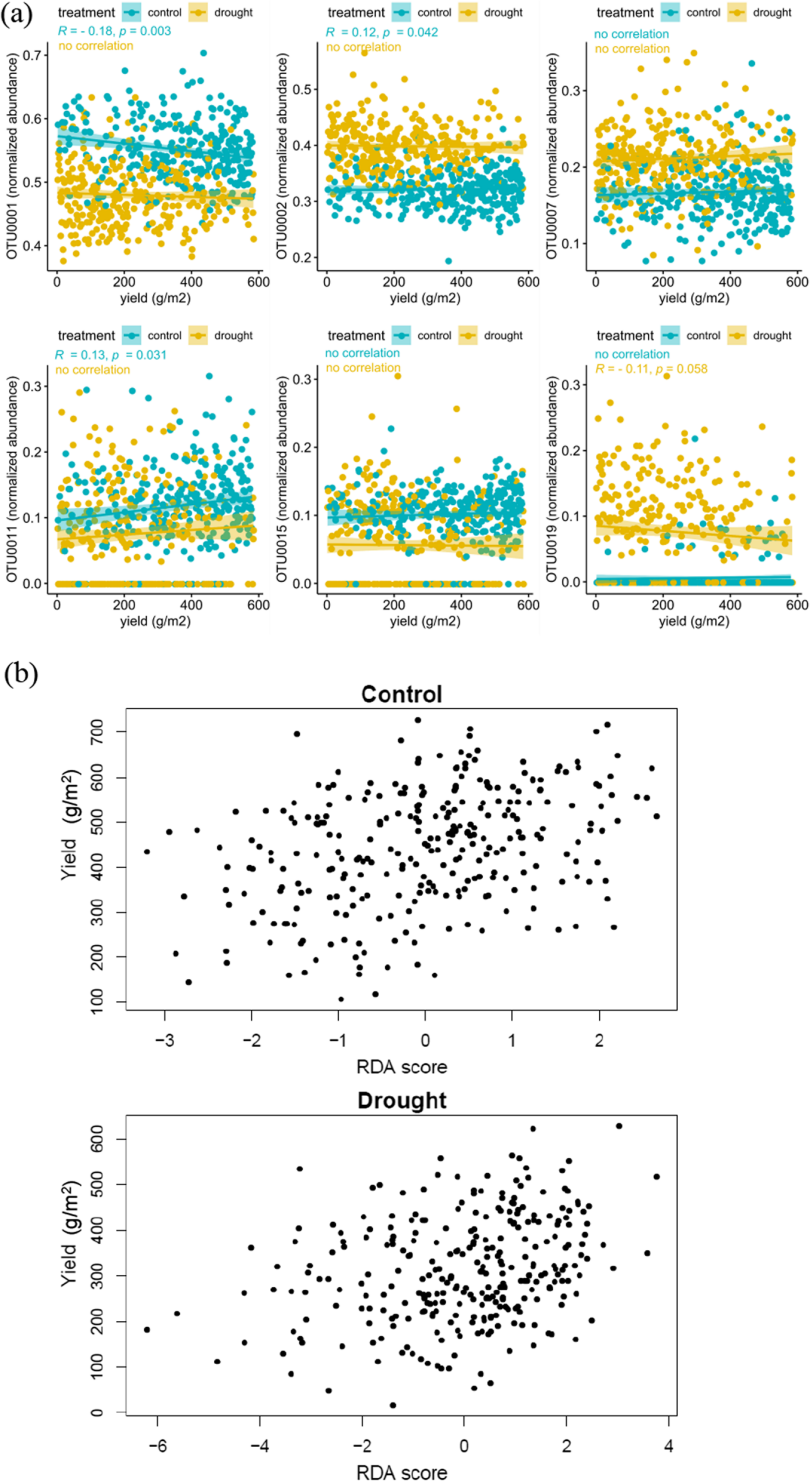
Effect of the sequence clusters on yield. **a** Correlation plots showing the relation between the abundance of sequence clusters and grain yield, for sequence clusters with at least one associated SNP. **b** RDA plot showing the relationship between the composition of the fungal community associated with the roots and yield. We analyzed control and drought samples separately as the structure of their microns differed substantially. Correlation per sequence-cluster with yield per treatment were tested with a permutation test. Each dot represents one sequence cluster community and its correlation with yield

To study to what extent the fungal microbial community correlates with plant performance, we first conducted a Spearman correlation analysis between the six sequence-clusters (identified above as being associated with genetic markers) and grain yield. The results showed that two sequence clusters (OTU_0002 *Ceratosphaeria* spp. and OTU_0011 *Boudiera* spp.) were positively correlated with yield under control conditions (*P* value < 0.01; R > 0.10), while two showed a negative correlation, one under control conditions (OTU_0001 Chaetomiaceae; *P* value = 0.003; R = − 0.18) and one under drought that was almost significant (OTU_0019 Rhizophydiales; *P* value = 0.058; R = − 0.11) (Fig. 2a). Interestingly, among the six sequence clusters, the one that correlated positively with yield under control conditions (OTU_0002) was less abundant under drought conditions (Fig. 1b, Additional file 1: Table S1).

A comprehensive correlation analysis of all sequence clusters and grain yield only resulted in eight significant correlations (*P* value < 0.05) (Additional file 1: Fig. S4a), and only under control conditions (Additional file 1: Table S2). To assess the multivariate effect of all the sequence clusters together on yield, we fitted an RDA with yield as constraining variable. The resulting constrained axis is a linear combination of the individual sequence clusters and represents the effect of all the taxa together on yield. For the control samples, yield as constraining variable tested significant (*P* value = 4e−04), for drought, the effect was smaller (*P* value = 0.076) (Fig. 2b). The higher the contribution to the yield axis (RDA score) the more independent the sequence-cluster yield effect. RDA analysis resulted in more sequence clusters that contribute to yield than in the correlation analysis used above (Additional file 1: Fig. S4). For all the sequence clusters that were found to be correlated with yield (i.e. correlation analysis) we also identified associated SNPs (Fig. 1, Additional file 1: Table S1), except for two sequence clusters (OTU_0015 and OTU_0019), both Rhizophydiales, that did not contribute to yield.

**Fig. 3:**
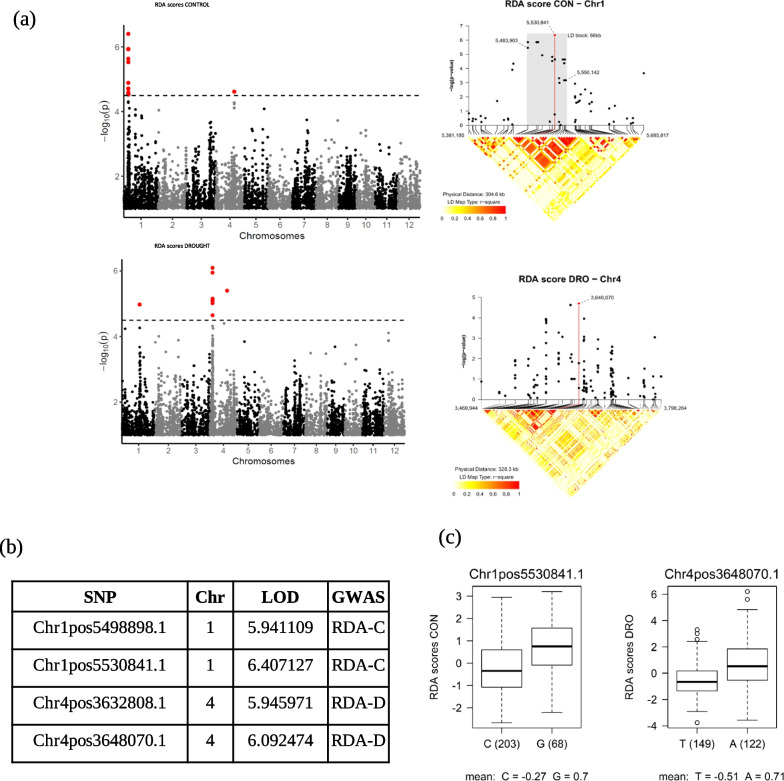
Host effect on yield-related fungal community. **a** Manhattan plots showing SNPs linked to RDA scores in the analysis of microbial community vs. yield. **b** List of SNPs associated with fungal sequence clusters. **c** Allelic effect of the two SNPs with the highest LOD score (one located on Chromosome 1 and the other on Chromosome 4)

The RDA scores were subsequently used for GWA analysis, and four SNPs were found to be significantly associated with the abundance of these yield-related sequence clusters (indirectly by their RDA scores), including eight SNPs on chromosome 1 under control conditions and seven SNPs, mainly on chromosome 4, under drought conditions (Fig. 3a, b). Among these SNPs, the one with the highest LOD score under the control treatment (Chr1pos5530841.1) and the one with the highest Lod score under drought treatment (Chr4pos3648070.1) had a clear allelic effect with a C/G and A/T change in chromosome 1 and 4, respectively (Fig. 3c).

### Gene Candidates

LD analysis with a window of 150 kb around each SNP was used to identify candidate genes (within chromosomal intervals) underlying the significant SNPs identified by GWA analysis, providing a list of a priori candidate genes that possibly control the association between root associated fungi and/or grain yield.

The list of candidate genes and their functional annotation was retrieved from the MSU Rice database (Table 2, Additional file 1: Table S1). When looking at the SNPs with the highest LOD score from the GWAS sequence-cluster (Additional file 1: Table S1), we identified candidate genes related to protease activity (serine protease SUBTILISIN, LOC_Os04g35140), cellulose synthesis protein (e.g. CELULOSE SYNTHASE-LIKE PROTEIN H2 (CSLH2) LOC_Os04g35020), anti-microbial defense (DEFENSIN-LIKE, DEFL, LOC_Os04g31250), Bric-à-brac/Tramtrack (BTB/POZ, LOC_Os10g28970) and EXO70 exocyst complex (LOC_Os04g31330), among other kinases and phosphatases (Table 1). The two SNPs with the highest LOD score from the GWAS with the RDA scores for sequence clusters associated with grain yield are linked to several candidate genes i.e. a peroxidase enzyme (LOC_Os04g06860), anti-microbial defense protein but on a different locus (*DEFENSIN-LIKE* (*DEFL),* LOC_Os01g10550) and cysteine-rich systemic response proteins (cysteine-rich *RAPID ALKALINIZATION FACTOR-LIKE* (*RALFL)*, LOC_Os01g10470) (under control conditions). These RALF proteins coordinate processes such as lateral root development and root hair growth, in response to environmental stimuli, e.g. plant hormones or pathogen infection (Campbell & Turner 2017) (Table 2). Several of these a priori candidate genes that are linked to microbial resistance, environmental responses, metabolite transport, and protease and protease inhibitors are promising candidates for involvement in microbiome recruitment and grain yield under drought.

**Table 1:** List of SNPs associated with fungal sequence clusters

Locus^a^	SNP	OTU	Taxonomy	OTU yield effect	Description	IC4R database*	RiceXPro database**
LOC_Os01g24060	Chr1pos13629118.1	OTU_0015	Cladochytriaceae fam	Positive	Importin subunit	FPKM < 1	− 3.5 (+ X.oryzae leaf)
LOC_Os02g43220	Chr2pos26097336.1	OTU_0002	Ceratosphaeria spp	Positive	Polyamine oxidase	FPKM < 1	1 (+ M.oryzae leaf) − 2.5 (+ ABA root); − 4
LOC_Os04g31410	Chr4pos18765756.1	OTU_0019	Rhizophydiales ord	Negative	RALFL	6 FPKM (root)	(+ JA root); − 3 (+ M.oryzae root)
LOC_Os04g35020	Chr4pos21345052.1	OTU_0011	Boudiera spp.	Positive	Cellulose synthase CSLH2	FPKM < 1	− 1 (+ M.oryzae root)
LOC_Os04g35060	Chr4pos21345052.1	OTU_0011	Boudiera spp.	Positive	Nicotinate phosphoribosyltransfer ase	20 FPKM (leaf); 14 FPKM (root)	5 (+ JA root); 1.5 (+ M.oryzae root)
LOC_Os04g35140	Chr4pos21345052.1	OTU_0011	Boudiera spp.	Positive	Subtilisin	3 FPKM (leaf); 5 FPKM (root)	1 (+ JA root); 3 (+ JA leaf); 2 (+ M.oryzae leaf); − 5 (+ X.oryzae leaf); − 2 (+ ABA root)
LOC_Os02g43250	Chr2pos26097336.1	OTU_0002	Ceratosphaeria spp	Positive	Kinase	7 FPKM (root, -Pi)	1 (+ ABA root); − 1.5 (+ M.oryzae root)
LOC_Os10g12620	Chr10pos7061662.1	OTU_0007	Cladosporium spp.	No effect	Kinase	4 FPKM (root)	No data
LOC_Os04g31250	Chr4pos18765756.1	OTU_0019	Rhizophydiales ord	Negative	Defensin (DEFL)	2 FPKM (leaf)	− 1 (+ JA root, leaf)
LOC_Os10g28970	Chr10pos15139435.1	OTU_0019	Rhizophydiales ord	Negative	Bric-a-brac/Tramtrack (BTB/POZ)	4 FPKM (root)	− 1 (+ JA root), − 2 (+ JA leaf), − 9 (+ X.oryzae leaf)
LOC_Os07g27580	Chr7pos16162723.1	OTU_0001	Chaetomium spp.	Negative	Haloacid dehalogenase (HAD) phosphatase	No data	No data
LOC_Os04g31330	Chr4pos18765756.1	OTU_0019	Rhizophydiales ord	Negative	Exo70 exocyst complex	20 FPKM (leaf)	− 1 (+ X.oryzae leaf)

Only SNPs where candidate genes were found within a 75 Kb window around the SNP are shown. All the SNPs were found in drought conditions, except the SNP related to OTU_0001 (LOC_Os07g27940) which was only found in the control treatment

^a^For some gene functions there are several loci found. Here only the most highlighted ones are represented

^*^Showing only expression values for leaf and root tissues. *FPKM:* Fragments Per Kilobase of transcript per Million mapped reads

^**^Showing Log_2_Fold expression values. + M.oryzae: inoculation with *Magnaporthe oryzae*; + X.oryzae: inoculation with *Xanthomonas oryzae*; + ABA: abscisic acid treatment; + JA: jasmonic acid treatment

**Table 2:** List of candidate genes associated with SNPs from the yield-related mycobiota

Locus^a^	SNP	RDA-GWAS	Description	IC4R database*	RiceXPro database**
LOC_Os01g10320	Chr1pos5498898.1	Control	HD-Zip protein	No data	No data
LOC_Os01g10350	Chr1pos5498898.1	Control	MPPN_protein	No data	No data
LOC_Os01g10370	Chr1pos5498898.1	Control	AP2 protein	1 FPKM (root)	4 (+ ABA leaf); 1 (+ JA leaf); − 2.5 (+ M.oryzae root/leaf)
LOC_Os01g10440	Chr1pos5498898.1	Control	Xylosyltransferase	FPKM < 1	4 (+ ABA root); 4 (+ JA root); 2.5 (+ M.oryzae root); 3.5 (+ M.oryzae leaf)
LOC_Os01g10450	Chr1pos5498898.1	Control	Kinase	no data	No data
LOC_Os01g10470	Chr1pos5530841.1	Control	RALFL17	FPKM < 1	No significant differences
LOC_Os01g10480	Chr1pos5530841.1	Control	Plastocyanin	FPKM < 1	No data
LOC_Os01g10490	Chr1pos5530841.1	Control	Keratin type I protein	No data	No data
LOC_Os01g10504	Chr1pos5530841.1	Control	OsMADS3	No data	No data
LOC_Os01g10550	Chr1pos5530841.1	Control	Defensin (DEFL)	FPKM < 1	No data
LOC_Os04g06790	Chr4pos3632808.1	Drought	Dioxygenase	14 FPKM (root)	No significant differences
LOC_Os04g06860	Chr4pos3632808.1	Drought	Peroxidase	No data	No data

^a^For some gene functions there are several loci found. Here only the most highlighted ones are represented

^*^Showing only expression values for leaf and root tissues. *FPKM:* Fragments Per Kilobase of transcript per Million mapped reads

^**^Showing Log_2_Fold expression values. + M.oryzae: inoculation with *Magnaporthe oryzae*; + X.oryzae: inoculation with *Xanthomonas oryzae*; + ABA: abscisic acid treatment; + JA: jasmonic acid treatment

## Discussion

Using a combination of high throughput sequencing of microbial marker genes and a bioinformatics pipeline, we characterized the composition of the fungal microbiome associated with rice roots under well-watered (control) and drought conditions. The use of an *indica* rice panel consisting of 247 genotyped accessions (Rebolledo et al. 2016; Kadam et al. 2017), representing a wide range of yield performance (Melandri et al. 2020a, b), allowed us to assess the diversity of rice-root mycobiota and to determine the extent to which this diversity is genetically controlled by the host plant, in both well-watered and drought conditions. Drought was shown to have a strong impact on the root mycobiota and to increase its mycobiota diversity (Additional file 1: Fig. S1b). The same was recently reported in a tropical grassland soil and in another study on rice (Andreo-Jimenez et al. 2019; Oliveira et al. 2020). Drought increased species diversity and evenness but at the same time reduced species richness compared to in water-sufficient conditions (Additional file 1: Fig. S1b). Despite the notable heterogeneity observed in plant-microbial communities of a single plant species grown on different soils, there is a growing body of evidence that the composition of plant microbiota is not randomly assembled but at least partially ruled by deterministic processes under the control of the host plant (Shi et al. 2018; Xiong et al. 2021). Accordingly, we identified ten SNPs associated with the abundance of specific fungal sequence clusters and four SNPs linked to sequence clusters correlated with yield. The candidate genes underlying these SNPs encode proteins related to microbial resistance, environmental responses, metabolite transport, and protease and protease inhibitors, and are promising candidates for involvement in the plant-microbiome interaction.

Increasing our knowledge of the plant-microbiome interaction with the final aim of enhancing agricultural productivity is a rapidly expanding research field. Little is known about how environmental changes like global warming and drought will affect root based microbial populations, and vice versa, how these microbial populations will contribute to resilience against these environmental challenges. The increase in root-associated fungal diversity under drought potentially equips the host with additional functions to mitigate the consequences of drought (van der Heijden & Hartmann 2016; Prudent et al. 2020). Pleosporales and Capnodiales, and to a lesser extent, Chaetothyriales and Rhizophydiales were more abundant under drought conditions, while Sordariales and Pezizales were more abundant under control conditions (Additional file 1: Fig. S1a). Pleosporales are ubiquitous fungi and include species with quite different functions, such as plant pathogens, saprophytes and plant endophytes, as well as plant beneficial fungi (Zhang et al. 2009). Inoculation of rice with members of the Pleosporales has been shown to improve nitrogen content and to boost plant growth (Vergara et al. 2019) and Pleosporales have been reported to be key taxa in the rice core seed microbiome (Eyre et al. 2019). Dark septate endophytes (DSE), which belong to Pleosporales, were reported to promote growth of the xerophyte, *Ammopiptanthus mongolicus*, under drought conditions (Li et al. 2018). A Pleosporales sequence cluster (OTU_0003) was shown to contribute most to grain yield under well-watered conditions (Additional file 1: Fig. S3), making this a highly interesting candidate fungus for follow-up research. Capnodiales, which were more abundant under drought in the present study?, are widespread and behave as saprotrophs, plant and human pathogens, mycoparasites and endophytes (Abdollahzadeh et al*.* 2020). For example, *Cladosporium fulvum* is a well-studied plant pathogen that causes tomato leaf mold (Thomma et al. 2005). In our study, a *Cladosporium* spp. sequence cluster contributed positively to yield under both drought and control treatments (Additional file 1: Figs. S1a, S4a). Species such as *Cladosporium cladosporioides* and *Cladosporium porophorum* are known fungal endophytes that can hyper-parasitize fungal pathogens of several crops, including rice (Becker et al. 2020; Chaibub et al. 2020; Erfandoust et al. 2020). Sordariales and Pezizales were more abundant in our control treatment. The former are saprotrophs, and plant pathogenic and wood inhabiting fungi. For instance, *Colletotrichum* spp. are serious plant pathogens that cause anthracnose and rotting diseases and affect a wide range of plant species (Cannon et al. 2012). Pezizales can be saprotrophs, ectomycorrhizae or pathogens in plants. Pezizomycetes, for example, occur in moist habitats in soil and in decomposing wood (Ekanayaka et al. 2018). In the present study, two sequence clusters belonging to Pezizales (*Boudiera acanthospora*) and Sordariales (*Ceratosphaeria lampadophora*) positively contributed to yield (Additional file 1: Fig. S3). *Ceratosphaeria* spp. are closely related to *Magnaporthe* spp. such as *M. grisea*, responsible for the rice blast, and occur widely in freshwater environments (Luo et al. 2019), but little is known concerning a potential beneficial role.

The positive effect of drought on microbial diversity has also been reported in artichoke in which rhizosphere bacterial diversity increased under salt stress (Yang et al. 2016). However, under long-term stress, diversity in ectomycorrhizal fungi in trees decreased, with a few beneficial species remaining as the dominant ones (Gehring et al. 2014). In our study, species richness under drought was lower, like in coastal plant populations where high salinity decreased AM fungi species richness (Guo & Gong 2014). The positive correlation of a number of sequence clusters with plant yield were dependent on the environment, with sequence clusters correlated with yield under either control or drought conditions (Fig. 2a; Additional file 1: Fig. S4). Positive correlations have also been reported for grapevine, in which drought can enhance the growth promoting effect of plant growth promoting bacteria, *Acinetobacter* and *Pseudomonas* (Rolli et al. 2015).

Ten different SNPs were found to be associated with the abundance of six independent sequence clusters, among which two were also correlated with grain yield under control conditions. We found several candidate genes, putatively involved in plant–microbe interactions, which may also be involved in sequence cluster abundance in rice roots, and possibly in rice yield. When zooming in on individual candidate genes, we found a number of candidate genes for disease resistance and plant development, including two that encode *RALF-LIKE* (*RALFL*) peptides, for instance. The RALF protein family is conserved across species and has a role in root development and as signaling molecules for stress adaptation responses (e.g. drought) (Sharma et al. 2016). Both *RALFL* candidate loci are downregulated in rice after root treatment with jasmonic acid (− 4 Log_2_Fold) and abscisic acid (− 4 Log_2_Fold), and *Magnaporthe oryzae* infection (− 3 Log_2_Fold) (data in the RiceXPro database: https://ricexpro.dna.affrc.go.jp/). RALF proteins are able to negatively regulate the plant immune system, and, in strawberry, have been shown to be differentially expressed upon inoculation with *Botrytis cinerea* (Negrini et al. 2020). In *Medicago truncatula*, RALF genes are induced by *nod* factors in the early steps of *Rhizobium* symbiosis (Kereszt et al*.*, 2018). Fungal pathogens like *Fusarium oxysporum* secrete RALF functional homologs, which was shown to facilitate their infection of roots (Masachis et al. 2016). The RALF genes are also of potential interest because of their role in root development (Sharma et al. 2016). Root developmental genes have been shown to shape the root associated fungal and bacterial microbiome in *Arabidopsis* (Bergelson et al. 2019), which supports the candidacy of this *RALFL* candidate gene for sequence cluster abundance in rice. In our study, the two *RALFL* loci were associated with a sequence cluster from the Rhizophydiales order that was slightly negatively correlated with yield under the drought treatment (Fig. 2). Rhizophydiales are diatom microparasites and are commonly found in water environments, and they compete with surrounding bacteria for microbial photosynthetic carbon, and consequently affect the composition of bacterial microbiome (Klawonn et al. 2021). We also found several candidate genes related to host–pathogen interactions and defense. One of them, defensin (DEFL), which appears in two loci (DEFL35 LOC_Os01g10550, DEFL49 LOC_Os04g31250), is a family of antimicrobial peptides that are involved in defense processes. In rice, two defensin proteins (OsDEF7 and OsDEF8) have been shown to act against two pathogenic bacteria, *Xanthomonas oryzae* and *Erwinia carotovora*, and OsAFP1 displayed antifungal activity against *Candida albicans* (Tantong et al. 2016; Ochiai et al. 2018). The defensin proteins found in the present study were associated with a fungus negatively correlated with yield (OTU_0019) and positively/negatively correlated with other yield related fungi under drought conditions (DEFL35). This suggests that fungal interactions affect yield more negatively in the rice plant i.e. OTU_0019. On the other hand, the expression pattern of DEFL proteins is more heterogenous than that of other pathogen defense related proteins such as NOD-like receptors (NLR), and DELF proteins display higher functional diversification i.e. development of reproductive organs, heavy metal resistance (Mondragón-Palomino et al. 2017). It would be interesting to create knock-out mutants of these DEFLs and allelic complementation lines to see what the consequences are for the recruitment of pathogenic and beneficial mycobiota. Another candidate gene, EXOCYST TETHERING COMPLEX (EXO70), is a regulator of secretor vesicles in the cell membrane, and these regulators are highly specialized. Exocyst tethering complex proteins are involved in several plant development processes, i.e. auxin-dependent root development, pollen maturation, germination, as well as in immune defense processes (Marković et al. 2021). For instance in rice, EXO70 is involved in defense against *Maganaporthe oryzae* (De la Concepcion et al. 2022). On the other hand, these EXO70 tethering complexes are also involved in symbiotic relationships. For instance, EXO70I is required for the development of the peri-arbuscular membrane during arbuscular mycorrhizal (AM) symbiosis in *Medicago truncatula* (Zhang et al. 2015; Ho-Plágaro et al. 2022). Furthermore, in legumes such as soybean, the tethering system GmExo70J is a precondition for establishment of the symbiosis with nitrogen-fixing rhizobia (Wang et al. 2016). Perhaps our EXO70 candidate gene in rice also plays a role in symbiosis with AM fungi, although, unexpectedly, it is highly expressed in rice leaves (Table 1). Finally, a large number of candidate genes found to be related to OTU_0019 under drought conditions belong to the Bric-a-Brac Tramtrack Broad/Poxvirus and Zinc finger (BTB/POZ) protein complex families that have a role in plant growth and development and plant defense regulation. Interestingly, BTB/POZ can stimulate but also repress the plant immune system (Orosa et al. 2017). For instance, in *Nicotiana benthamiana,* the BTB/POZ complex NbBTB has been shown to negatively regulate plant resistance against *Phytophthora parasitica* and is easily triggered by pathogen effectors (Zhao et al. 2022).

Among all the fungal-mediated yield SNPs found in our study, a clear difference was evidenced between drought and control, with SNPs on chromosome 1 more present under control conditions and SNPs on chromosome 4 more present under drought conditions (Fig. 3). These sequence clusters are interesting molecular targets and the underlying genes could be involved in plant–microbe symbioses as well as in the combined response of the plant-mycobiota and its impact on plant fitness under stressful conditions such as drought, and yield under control conditions. Interestingly among the candidate genes that influence the abundance of yield related sequence clusters, we found some that encode disease resistance genes (*DEFL*). As discussed above, receptors related to host defense and symbiosis are structurally similar, making them interesting candidate genes for resistance.

## Conclusions

Our study provides new candidate genes in rice that may be involved in interactions between fungi and rice roots, including both pathogens and mutualistic fungi. Moreover, some of the candidate genes are related to abiotic stress responses especially sequence clusters that are correlated with yield. The genes responsible for the abundance/presence of independent sequence-clusters may indirectly contribute to the plant phenotype in response to drought stress. The candidate genes should be further investigated in follow-up experiments to see how robust their association is with the trait of interest. Starting from a fungal culture collection isolated from these rice cultivars, experimental inoculation with the fungal isolates detected in the GWAS analysis would make it possible to test their effect on rice drought tolerance and on yield. Our study also opens up new avenues for further analyses of the mechanisms underlying the plant-root-mycobiota interaction and drought tolerance. This information would be extremely valuable for rice breeding programs aimed at developing genotypes that are better equipped to recruit beneficial fungi (or to avoid pathogenic fungi) that will help increase yields under sub-optimal conditions, such as drought.

## Supplementary Information

**Additional file 1**. **Supplementary Tables and Figures. Fig. S1:** Fungal community description. **Fig. S2:** PCA of the mycobiota. **Fig. S3:** Boxplots with SNP allelic effect. **Fig. S4:** Sequence-clusters contributing to plant yield. **Table S1:** List of SNPs found to be associated with specific fungal-clusters abundances and with drought-related fungalclusters. **Table S2:** Sequence-clusters contributing to plant yield. **Fig. S5:** Mantel Test plots. **Fig. S6:** Mycobiota Phyla abundances.

**Additional file 2**. **Supplementary Data File.** It contains taxonomic information and abundance of the most abundant sequence-clusters (OTUs) used for the GWAS analysis (n = 80), their RDA scores, SNPs found and candidate genes list. OTU selection: sequence-clusters that occurred at least 25 times with a total number of reads of at least 100. This was done separately for drought and control.

## Data Availability

Supplementary figures and tables are included as additional files. The rest of supporting information and data will is  available in the 4TU. ResearchData database, 10.4121/9526a9b6-0690-4829-aed5-e4b88734e6c1.

## Abbreviations

GWAS: Genome wide association study
OTU: Operational taxonomic unit/sequence cluster
SNP: Single nucleotide polymorphism
RDA: Redundancy analysis
DEFL: Defensin-like protein
RALFL: Rapid alkalinization factor-like protein
EXO: Exocyst tethering complex
BTB/POZ: Bric-a-Brac tramtrack broad/poxvirus and zinc finger complex

## References

[CR1] Abedini D, Jaupitre S, Bouwmeester H, Dong L (2021). Metabolic interactions in beneficial microbe recruitment by plants. Curr Opin Biotechnol.

[CR300] Abdollahzadeh J, Groenewald JZ, Coetzee MPA, Wingfield MJ, Crous PW. (2020) Evolution of lifestyles in Capnodiales. Stud Mycol 95:381–414. 10.1016/j.simyco.2020.02.004 10.1016/j.simyco.2020.02.004 PMC7426231 32855743

[CR2] Andreo-Jimenez B, Vandenkoornhuyse P, Van AL, Heutinck A, Duhamel M, Kadam N, Jagadish K, Ruyter-Spira C, Bouwmeester H (2019). Plant host and drought shape the root associated fungal microbiota in rice. PeerJ.

[CR3] Azad K, Kaminskyj S (2015). A fungal endophyte strategy for mitigating the effect of salt and drought stress on plant growth. Symbiosis.

[CR4] Becker R, Ulrich K, Behrendt U, Kube M, Ulrich A (2020). Analyzing ash leaf-colonizing fungal communities for their biological control of hymenoscyphus fraxineus. Front Microbiol.

[CR5] Beilsmith K, Thoen MPM, Brachi B, Gloss AD, Khan MH, Bergelson J (2019). Genome-wide association studies on the phyllosphere microbiome: embracing complexity in host–microbe interactions. Plant J.

[CR6] Bergelson J, Mittelstrass J, Horton MW (2019). Characterizing both bacteria and fungi improves understanding of the Arabidopsis root microbiome. Sci Rep.

[CR7] Bernaola L, Cosme M, Schneider RW, Stout M (2018). Belowground inoculation with arbuscular mycorrhizal fungi increases local and systemic susceptibility of rice plants to different pest organisms. Front Plant Sci.

[CR8] Borneman J, Hartin RJ (2000). PCR primers that amplify fungal rRNA genes from environmental samples. Appl Environ Microbiol.

[CR9] Brachi B, Morris GP, Borevitz JO (2011). Genome-wide association studies in plants: the missing heritability is in the field. Genome Biol.

[CR10] Brown SP, Grillo MA, Podowski JC, Heath KD (2020). Soil origin and plant genotype structure distinct microbiome compartments in the model legume Medicago truncatula. Microbiome.

[CR11] Callahan BJ, McMurdie PJ, Holmes SP (2017). Exact sequence variants should replace operational taxonomic units in marker-gene data analysis. ISME J.

[CR12] Camacho C, Coulouris G, Avagyan V, Ma N, Papadopoulos J, Bealer K, Madden TL (2009). BLAST+: architecture and applications. BMC Bioinformatics.

[CR13] Campbell L, Turner SR (2017). A Comprehensive analysis of ralf proteins in green plants suggests there are two distinct functional groups. Front Plant Sci.

[CR14] Cannon PF, Damm U, Johnston PR, Weir BS (2012). Colletotrichum – current status and future directions. Stud Mycol.

[CR15] Cattivelli L, Rizza F (2008). Drought tolerance improvement in crop plants: an integrated view from breeding to genomics. Field Crops Res.

[CR16] Chadha N, Mishra M, Rajpal K, Bajaj R, Choudhary DK, Varma A (2015). An ecological role of fungal endophytes to ameliorate plants under biotic stress. Arch Microbiol.

[CR17] Chaibub AA, de Sousa TP, de Araújo LG, de Filippi MCC (2020). Cladosporium cladosporioides C24G modulates gene expression and enzymatic activity during leaf blast suppression in rice plants. J Plant Growth Regul.

[CR18] Choi J, Lee T, Cho J, Servante EK, Pucker B, Summers W, Bowden S, Rahimi M, An K, An G (2020). The negative regulator SMAX1 controls mycorrhizal symbiosis and strigolactone biosynthesis in rice. Nat Commun.

[CR19] Dastogeer KMG, Li H, Sivasithamparam K, Jones MGK, Wylie SJ (2018). Fungal endophytes and a virus confer drought tolerance to Nicotiana benthamiana plants through modulating osmolytes, antioxidant enzymes and expression of host drought responsive genes. Environ Exp Bot.

[CR20] De la Concepcion JC, Fujisaki K, Bentham AR, Cruz Mireles N, de Medina S, Hernandez V, Shimizu M, Lawson DM, Kamoun S, Terauchi R, Banfield MJ (2022). A blast fungus zinc-finger fold effector binds to a hydrophobic pocket in host Exo70 proteins to modulate immune recognition in rice. Proc Natl Acad Sci USA.

[CR21] de Oliveira TB, de Lucas RC, de Scarcella AS (2020). Fungal communities differentially respond to warming and drought in tropical grassland soil. Mol Ecol.

[CR22] Deng S, Caddell DF, Xu G, Dahlen L, Washington L, Yang J, Coleman-Derr D (2021). Genome wide association study reveals plant loci controlling heritability of the rhizosphere microbiome. ISME J.

[CR23] Duc NH, Csintalan Z, Posta K (2018). Arbuscular mycorrhizal fungi mitigate negative effects of combined drought and heat stress on tomato plants. Plant Physiol Biochem.

[CR24] Edwards J, Johnson C, Santos-Medellín C, Lurie E, Podishetty NK, Bhatnagar S, Eisen JA, Sundaresan V (2015). Structure, variation, and assembly of the root-associated microbiomes of rice. Proc Natl Acad Sci.

[CR25] Ekanayaka AH, Hyde KD, Jones EBG, Zhao Q (2018). Taxonomy and phylogeny of operculate discomycetes: pezizomycetes. Fungal Diversity.

[CR26] Erfandoust R, Habibipour R, Soltani J (2020). Antifungal activity of endophytic fungi from cupressaceae against human pathogenic aspergillus fumigatus and aspergillus niger. J De Mycol Méd.

[CR27] Escudero-Martinez C, Coulter M, Terrazas RA, Foito A, Kapadia R, Pietrangelo L, Maver M, Sharma R, Aprile A, Morris J (2022). Identifying plant genes shaping microbiota composition in the barley rhizosphere. Nat Comun.

[CR28] Escudié F, Auer L, Bernard M, Mariadassou M, Cauquil L, Vidal K, Maman S, Hernandez-Raquet G, Combes S, Pascal G (2018). FROGS: find, rapidly, OTUs with galaxy solution. Bioinformatics.

[CR29] Eyre AW, Wang M, Oh Y, Dean RA (2019). Identification and characterization of the core rice seed microbiome. Phytobiomes J.

[CR30] Famoso AN, Zhao K, Clark RT, Tung C-W, Wright MH, Bustamante C, Kochian LV, McCouch SR (2011). Genetic architecture of aluminum tolerance in rice (*Oryza sativa*) determined through genome-wide association analysis and QTL mapping. PLoS Genet.

[CR31] Fox J, Weisberg S (2011). An R companion to applied regression.

[CR32] Gehring C, Flores-Rentería D, Sthultz CM, Leonard TM, Flores-Rentería L, Whipple AV, Whitham TG (2014). Plant genetics and interspecific competitive interactions determine ectomycorrhizal fungal community responses to climate change. Mol Ecol.

[CR33] Geisen S, ten Hooven FC, Kostenko O, Snoek LB, van der Putten WH (2021). Fungal root endophytes influence plants in a species-specific manner that depends on plant’s growth stage. J Ecol.

[CR34] Guo X, Gong J (2014). Differential effects of abiotic factors and host plant traits on diversity and community composition of root-colonizing arbuscular mycorrhizal fungi in a salt-stressed ecosystem. Mycorrhiza.

[CR35] Han B, Huang X (2013). Sequencing-based genome-wide association study in rice. Curr Opin Plant Biol.

[CR36] Hardoim PR, van Overbeek LS, Berg G, Pirttilä AM, Compant S, Campisano A, Döring M, Sessitsch A (2015). The hidden world within plants: ecological and evolutionary considerations for defining functioning of microbial endophytes. Microbiol Mol Biol Rev.

[CR37] Ho-Plágaro T, Tamayo-Navarrete MI, García Garrido JM (2022). Microtubule cytoskeleton and mycorrhizal roots. Plant Signal Behav.

[CR38] Horton MW, Bodenhausen N, Beilsmith K, Meng D, Muegge BD, Sathish Subramanian M, Vetter M, Vilhjálmsson BJ, Nordborg M, Gordon JI, Bergelson J (2014). Genome-wide association study of Arabidopsis thaliana leaf microbial community. Nat Commun.

[CR39] Huang X, Wei X, Sang T, Zhao Q, Feng Q, Zhao Y, Li C, Zhu C, Lu T, Zhang Z (2010). Genome-wide association studies of 14 agronomic traits in rice landraces. Nat Genet.

[CR40] Kadam NN, Tamilselvan A, Lawas LMF, Quinones C, Bahuguna RN, Thomson MJ, Dingkuhn M, Muthurajan R, Struik PC, Xinyou Yin SV, Jagadish K (2017). Genetic control of plasticity in root morphology and anatomy of rice in response to water deficit. Plant Physiol.

[CR200] Kadam NN, Struik PC, Rebolledo MC, Yin X, Jagadish SVK (2018) Genome-wide association reveals novel genomic loci controlling rice grain yield and its component traits under water-deficit stress during the reproductive stage. J Experiment Botany 69:4017–4032 10.1093/jxb/ery186 PMC6054195 29767744

[CR41] Kang HM (2010). Variance component model to account for sample structure in genome-wide association studies. Nat Genet.

[CR400] Kereszt A, Mergaert P, Montiel J, Endre G and Kondorosi É (2018) Impact of plant peptides on symbiotic nodule development and functioning. Front Plant Sci 9:1026. 10.3389/fpls.2018.01026 10.3389/fpls.2018.01026 PMC6056668 30065740

[CR42] Kim B, Westerhuis JA, Smilde AK, Floková K, Suleiman AKA, Kuramae EE, Bouwmeester HJ, Zancarini A (2022). Effect of strigolactones on recruitment of the rice root-associated microbiome. FEMS Microbiol Ecol.

[CR43] Kindt R, Coe R (2005). Tree diversity analysis: a manual and software for common statistical methods for ecological and biodiversity studies.

[CR44] Klawonn I, Van den Wyngaert S, Parada AE, Arandia-Gorostidi N, Whitehouse MJ, Grossart H-P, Dekas AE (2021). Characterizing the “fungal shunt”: parasitic fungi on diatoms affect carbon flow and bacterial communities in aquatic microbial food webs. Proc Natl Acad Sci.

[CR45] Kozich JJ, Westcott SL, Baxter NT, Highlander SK, Schloss PD (2013). Development of a dual-index sequencing strategy and curation pipeline for analyzing amplicon sequence data on the MiSeq illumina sequencing platform. Appl Environ Microbiol.

[CR46] Kruijer W, Boer MP, Malosetti M, Flood PJ, Engel B, Kooke R, Keurentjes JJB, van Eeuwijk FA (2015). Marker-based estimation of heritability in immortal populations. Genetics.

[CR47] Kuschel-Otárola M, Rivera D, Holzapfel E, Schütze N, Neumann P, Godoy-Faúndez A (2020). Simulation of water-use efficiency of crops under different irrigation strategies. Water.

[CR48] Leopold DR, Busby PE (2020). Host genotype and colonist arrival order jointly govern plant microbiome composition and function. Curr Biol.

[CR49] Li X, He X, Hou L, Ren Y, Wang S, Su F (2018). Dark septate endophytes isolated from a xerophyte plant promote the growth of Ammopiptanthus mongolicus under drought condition. Sci Rep.

[CR50] Ling X, Naylor D, Dong Z, Simmons T, Pierroz G, Hixson KK, Kim Y-M, Zink EM, Engbrecht KM, Wang Y, Gao C, DeGraaf S, Madera MA, Sievert JA, Hollingsworth J, Birdseye D, Scheller HV, Hutmacher R, Dahlberg J, Jansson C, Taylor JW, Lemaux PG, Coleman-Derr D (2018). Drought delays development of the sorghum root microbiome and enriches for monoderm bacteria. Proc Natl Acad Sci.

[CR51] Liu J, Zehnder AJB, Yang H (2009). Global consumptive water use for crop production: the importance of green water and virtual water. Water Resour Res.

[CR52] Liu F, Hewezi T, Lebeis SL, Pantalone V, Grewal PS, Staton ME (2019). Soil indigenous microbiome and plant genotypes cooperatively modify soybean rhizosphere microbiome assembly. BMC Microbiol.

[CR53] Luo Z-L, Hyde KD, Liu J-K (2019). Freshwater sordariomycetes. Fungal Divers.

[CR54] Maciá-Vicente JG, Jansson H-B, Mendgen K, Lopez-Llorca LV (2008). Colonization of barley roots by endophytic fungi and their reduction of take-all caused by Gaeumannomyces graminis var. tritici. Can J Microbiol.

[CR55] Marković V, Kulich I, Žárský V (2021). Functional Specialization within the EXO70 gene family in Arabidopsis. Int J Mol Sci.

[CR56] Martin M (2011). Cutadapt removes adapter sequences from high-throughput sequencing reads. Embnet.j.

[CR57] Masachis S, Segorbe D, Turrà D, Leon-Ruiz M, Fürst U, El Ghalid M, Leonard G, López-Berges MS, Richards TA, Felix G (2016). A fungal pathogen secretes plant alkalinizing peptides to increase infection. Nat Microbiol.

[CR58] Mather KA, Caicedo AL, Polato NR, Olsen KM, McCouch S, Purugganan MD (2007). The extent of linkage disequilibrium in rice (*Oryza sativa L.*). Genetics.

[CR59] McCouch SR, Wright MH, Tung C-W, Maron LG, McNally KL, Fitzgerald M, Singh N, DeClerck G, Agosto-Perez F, Korniliev P (2016). Open access resources for genome-wide association mapping in rice. Nat Commun.

[CR60] Mejía LC, Rojas EI, Maynard Z, Bael SV, Arnold AE, Hebbar P, Samuels GJ, Robbins N, Herre EA (2008). Endophytic fungi as biocontrol agents of *Theobroma cacao* pathogens. Biol Control.

[CR61] Melandri G, AbdElgawad H, Riewe D, Hageman JA, Asard H, Beemster GTS, Kadam N, Jagadish K, Altmann T, Ruyter-Spira C (2020). Biomarkers for grain yield stability in rice under drought stress. J Exp Bot.

[CR62] Melandri G, Prashar A, McCouch SR, van der Linden G, Jones HG, Kadam N, Jagadish K, Bouwmeester H, Ruyter-Spira C (2020). Association mapping and genetic dissection of drought-induced canopy temperature differences in rice. J Exp Bot.

[CR63] Melandri G, Sikirou M, Arbelaez JD, Shittu A, Semwal VK, Konaté KA, Maji AT, Ngaujah SA, Akintayo I, Govindaraj V (2021). Multiple small-effect alleles of Indica origin enhance high iron-associated stress tolerance in rice under field conditions in West Africa. Front Plant Sci.

[CR64] Mondragón-Palomino M, Stam R, John-Arputharaj A, Dresselhaus T (2017). Diversification of defensins and NLRs in arabidopsis species by different evolutionary mechanisms. BMC Evol Biol.

[CR65] Negrini F, O’Grady K, Hyvönen M, Folta KM, Baraldi E (2020). Genomic structure and transcript analysis of the rapid alkalinization factor (RALF) gene family during host-pathogen crosstalk in Fragaria vesca and Fragaria x ananassa strawberry. PLoS ONE.

[CR66] Ochiai A, Ogawa K, Fukuda M, Ohori M, Kanaoka T, Tanaka T, Taniguchi M, Sagehashi Y (2018). Rice defensin OsAFP1 is a new drug candidate against human pathogenic fungi. Sci Rep.

[CR67] Oksanen J, Blanchet FG, Kindt R, Legendre P, Minchin PR, O’Hara RB, Simpson GL, Solymos P, Stevens MHL, Wagner H. 2015. Package ‘vegan’: community ecology package, version 2.3–2.

[CR68] Orosa B, He Q, Mesmar J, Gilroy EM, McLellan H, Yang C, Craig A, Bailey M, Zhang C, Moore JD (2017). BTB-BACK domain protein POB1 suppresses immune cell death by targeting ubiquitin E3 ligase PUB17 for degradation. PLoS Genet.

[CR69] Oyserman BO, Flores SS, Griffioen T, Pan X, van der Wijk E, Pronk L, Lokhorst W, Nurfikari A, Paulson JN, Movassagh M (2022). Disentangling the genetic basis of rhizosphere microbiome assembly in tomato. Nat Commun.

[CR70] Pandey V, Shukla A (2015). Acclimation and tolerance strategies of rice under drought stress. Rice Sci.

[CR71] Prudent M, Dequiedt S, Sorin C, Girodet S, Nowak V, Duc G, Salon C, Maron P-A (2020). The diversity of soil microbial communities matters when legumes face drought. Plant Cell Environ.

[CR201] R Core Team (2021) R: A language and environment for statistical computing. R Foundation for Statistical Computing, Vienna, Austria. URL https://www.R-project.org/

[CR72] Rebolledo MC, Peña AL, Duitama J, Cruz DF, Dingkuhn M, Grenier C, Tohme J (2016). Combining image analysis, genome wide association studies and different field trials to reveal stable genetic regions related to panicle architecture and the number of spikelets per panicle in rice. Front Plant Sci.

[CR73] Redman RS, Kim YO, Woodward CJDA, Greer C, Espino L, Doty SL, Rodriguez RJ (2011). Increased fitness of rice plants to abiotic stress via habitat adapted symbiosis: a strategy for mitigating impacts of climate change. PLoS ONE.

[CR74] Ritchie ME, Phipson B, Wu D, Hu Y, Law CW, Shi W, Smyth GK (2015). limma powers differential expression analyses for RNA-sequencing and microarray studies. Nucleic Acids Res.

[CR75] Rodriguez RJ, Henson J, Van Volkenburgh E, Hoy M, Wright L, Beckwith F, Kim Y-O, Redman RS (2008). Stress tolerance in plants via habitat-adapted symbiosis. ISME J.

[CR76] Rolli E, Marasco R, Vigani G, Ettoumi B, Mapelli F, Deangelis ML, Gandolfi C, Casati E, Previtali F, Gerbino R (2015). Improved plant resistance to drought is promoted by the root-associated microbiome as a water stress-dependent trait. Environ Microbiol.

[CR77] Roman-Reyna V, Pinili D, Borja FN, Quibod IL, Groen SC, Alexandrov N, Mauleon R, Oliva R (2020). Characterization of the Leaf microbiome from whole-genome sequencing data of the 3000 rice genomes project. Rice.

[CR78] Ruiz-Sánchez M, Aroca R, Muñoz Y, Polón R, Ruiz-Lozano JM (2010). The arbuscular mycorrhizal symbiosis enhances the photosynthetic efficiency and the antioxidative response of rice plants subjected to drought stress. J Plant Physiol.

[CR79] Salvioli A, Bonfante P (2013). Systems biology and ‘omics’ tools: a cooperation for next-generation mycorrhizal studies. Plant Sci.

[CR80] Santos-Medellín C, Edwards J, Liechty Z, Nguyen B, Sundaresan V (2017). Drought stress results in a compartment-specific restructuring of the rice root-associated microbiomes. Mbio.

[CR81] Schwender H, Li Q, Neumann C, Taub MA, Younkin SG, Berger P, Scharpf RB, Beaty TH, Ruczinski I (2014). Dectecting disease variants in case-parent trio studies using the Bioconductor software package trio. Genet Epidemiol.

[CR82] Sessitsch A, Hardoim P, Döring J, Weilharter A, Krause A, Woyke T, Mitter B, Hauberg-Lotte L, Friedrich F, Rahalkar M, Hurek T, Sarkar A, Bodrossy L, van Overbeek L, Brar D, van Elsas JD, Reinhold-Hurek B (2012). Functional characteristics of an endophyte community colonizing rice roots as revealed by metagenomic analysis. Mol Plant-Microbe Interact.

[CR83] Sharma A, Hussain A, Mun B-G, Imran QM, Falak N, Lee S-U, Kim JY, Hong JK, Loake GJ, Ali A (2016). Comprehensive analysis of plant rapid alkalization factor (RALF) genes. Plant Physiol Biochem.

[CR84] Shi Y, Li Y, Xiang X, Sun R, Yang T, He D, Zhang K, Ni Y, Zhu Y-G, Adams JM (2018). Spatial scale affects the relative role of stochasticity versus determinism in soil bacterial communities in wheat fields across the North China Plain. Microbiome.

[CR85] Simmons T, Styer AB, Pierroz G, Gonçalves AP, Pasricha R, Hazra AB, Bubner P, Coleman-Derr D (2020). Drought drives spatial variation in the millet root microbiome. Front Plant Sci.

[CR86] Tantong S, Pringsulaka O, Weerawanich K, Meeprasert A, Rungrotmongkol T, Sarnthima R, Roytrakul S, Sirikantaramas S (2016). Two novel antimicrobial defensins from rice identified by gene coexpression network analyses. Peptides.

[CR87] Thomma BPHJ, Esse HPV, Crous PW, Wit PJGMD (2005). Cladosporium fulvum (syn. Passalora fulva), a highly specialized plant pathogen as a model for functional studies on plant pathogenic Mycosphaerellaceae. Mol Plant Pathol.

[CR88] Trenberth KE, Dai A, van der Schrier G, Jones PD, Barichivich J, Briffa KR, Sheffield J (2014). Global warming and changes in drought. Nat Clim Chang.

[CR89] van der Heijden MGA, Hartmann M (2016). Networking in the plant microbiome. PLOS Biol.

[CR90] Vandenkoornhuyse P, Quaiser A, Duhamel M, Le Van A, Dufresne A (2015). The importance of the microbiome of the plant holobiont. New Phytol.

[CR91] Vergara C, Araujo KEC, Sperandio MVL, Santos LA, Urquiaga S, Zilli JÉ (2019). Dark septate endophytic fungi increase the activity of proton pumps, efficiency of 15N recovery from ammonium sulphate, N content, and micronutrient levels in rice plants. Braz J Microbiol.

[CR92] Wagner MR, Lundberg DS, del Rio TG, Tringe SG, Dangl JL, Mitchell-Olds T (2016). Host genotype and age shape the leaf and root microbiomes of a wild perennial plant. Nat Commun.

[CR93] Wallace JG, Kremling KA, Kovar LL, Buckler ES (2018). Quantitative genetics of the maize leaf microbiome. Phytobiomes Journal.

[CR94] Wang Z, Li P, Yang Y, Chi Y, Fan B, Chen Z (2016). Expression and functional analysis of a novel group of legume-specific WRKY and Exo70 protein variants from soybean. Sci Rep.

[CR95] Worchel ER, Giauque HE, Kivlin SN (2012). Fungal symbionts alter plant drought response. Microb Ecol.

[CR96] Xiong C, Zhu Y-G, Wang J-T, Singh B, Han L-L, Shen J-P, Li P-P, Wang G-B, Wu C-F, Ge A-H (2021). Host selection shapes crop microbiome assembly and network complexity. New Phytol.

[CR97] Yang H, Hu J, Long X, Liu Z, Rengel Z (2016). Salinity altered root distribution and increased diversity of bacterial communities in the rhizosphere soil of Jerusalem artichoke. Sci Rep.

[CR98] Yuan Z, Zhang C, Lin F, Kubicek CP (2010). Identity, diversity, and molecular phylogeny of the endophytic mycobiota in the roots of rare wild rice (*Oryza granulate*) from a nature reserve in Yunnan, China. Appl Environ Microbiol.

[CR99] Zargar SM, Raatz B, Sonah H, MuslimaNazir BJA, Dar ZA, Agrawal GK, Rakwal R (2015). Recent advances in molecular marker techniques: insight into QTL mapping, GWAS and genomic selection in plants. J Crop Sci Biotechnol.

[CR100] Zhang S-W, Li C-H, Cao J, Zhang Y-C, Zhang S-Q, Xia Y-F, Sun D-Y, Sun Y (2009). Altered architecture and enhanced drought tolerance in rice via the down-regulation of indole-3-acetic acid by TLD1/OsGH3.13 activation. Plant Physiol.

[CR101] Zhang X, Pumplin N, Ivanov S, Harrison MJ (2015). EXO70I Is required for development of a sub-domain of the periarbuscular membrane during arbuscular mycorrhizal symbiosis. Current Biol CB.

[CR102] Zhao K, Tung CW, Eizenga GC, Wright MH, Ali ML, Price AH, Norton GJ, Islam MR, Reynolds A, Mezey J (2011). Genome-wide association mapping reveals a rich genetic architecture of complex traits in Oryza sativa. Nat Commun.

[CR103] Zhao M, Ge Y, Xu Z, Ouyang X, Jia Y, Liu J, Zhang M, An Y (2022). A BTB/POZ domain-containing protein negatively regulates plant immunity in Nicotiana benthamiana. Biochem Biophys Res Commun.

