# Supplementary material for: Genetic Mapping of the Root Mycobiota in Rice and its Role in Drought Tolerance
Source: Rice (N Y). 2023 May 22;16:26. doi: 10.1186/s12284-023-00641-4 (PMC10203090; doi:10.1186/s12284-023-00641-4)

Additional File 1

Fig. S1 Mycobiota description. (a) Mycobiota found in the study. Bars represent total sum of abundances for each taxa. Order level is represented. Total number of sequence-clusters in each treatment is represented in the Venn diagram. (b) Description of the community per treatment. Means and SD shown. ANOVA shows that all parameters (Shannon, richness, J-eveness and abundance) are significantly different between treatments (*P* > 0.001).

(a)

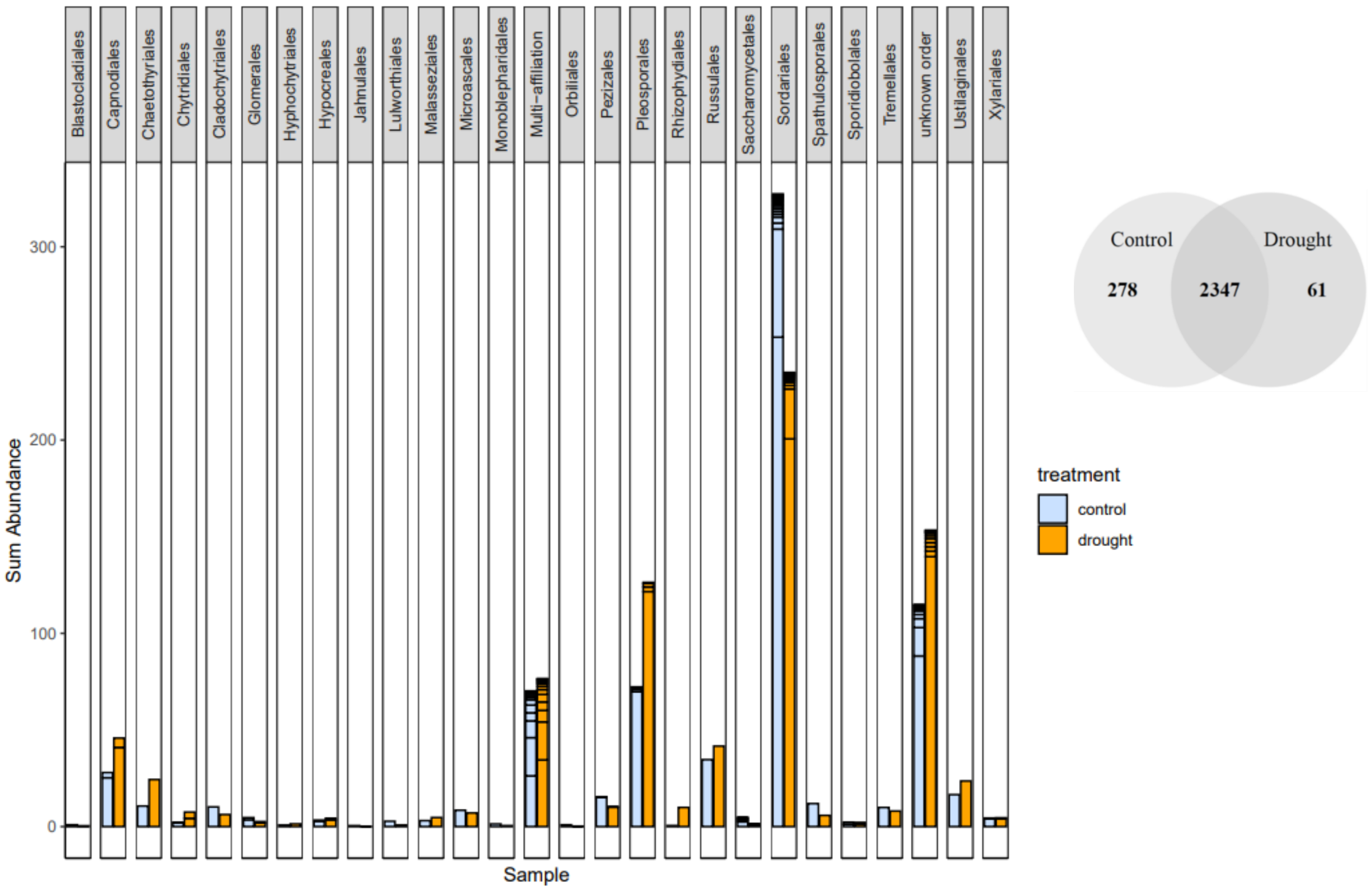

(b)

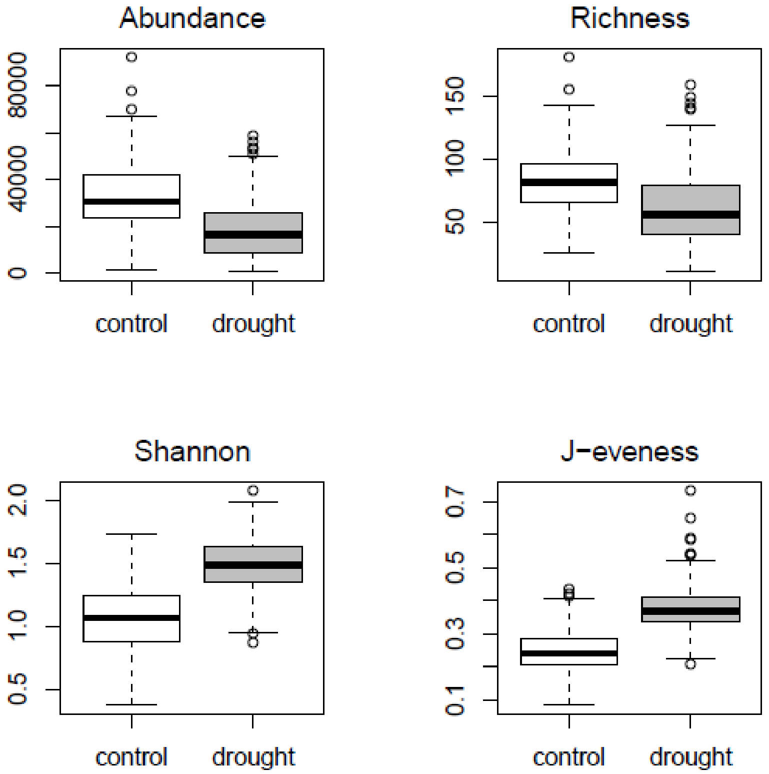

Abundance
0
40000
80000
control
drought
Richness
50
100
150
control
drought
Shannon
0.5
1.0
1.5
2.0
control
drought
J−eveness
0.1
0.3
0.5
0.7
control
drought

Fig. S2 PCA of the mycobiota. Sequence-clusters community in control (blue) and drought (red). Data was transformed with log-proportions and corrected for the sequencing run plate effect before performing the PCA analysis.

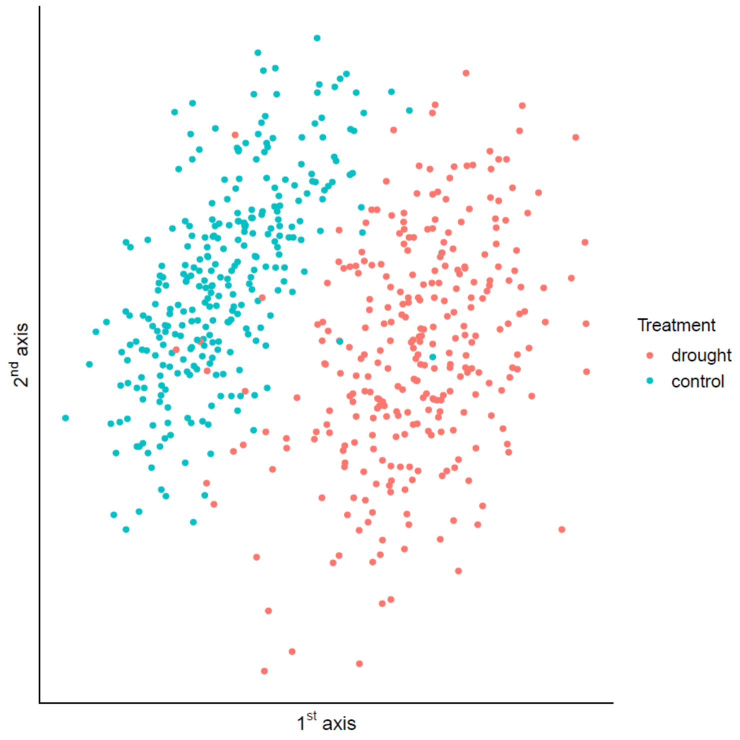

Fig. S3 Boxplots with SNP allelic effect. All SNPs found in the univariate GWAS with sequence-clusters are shown.

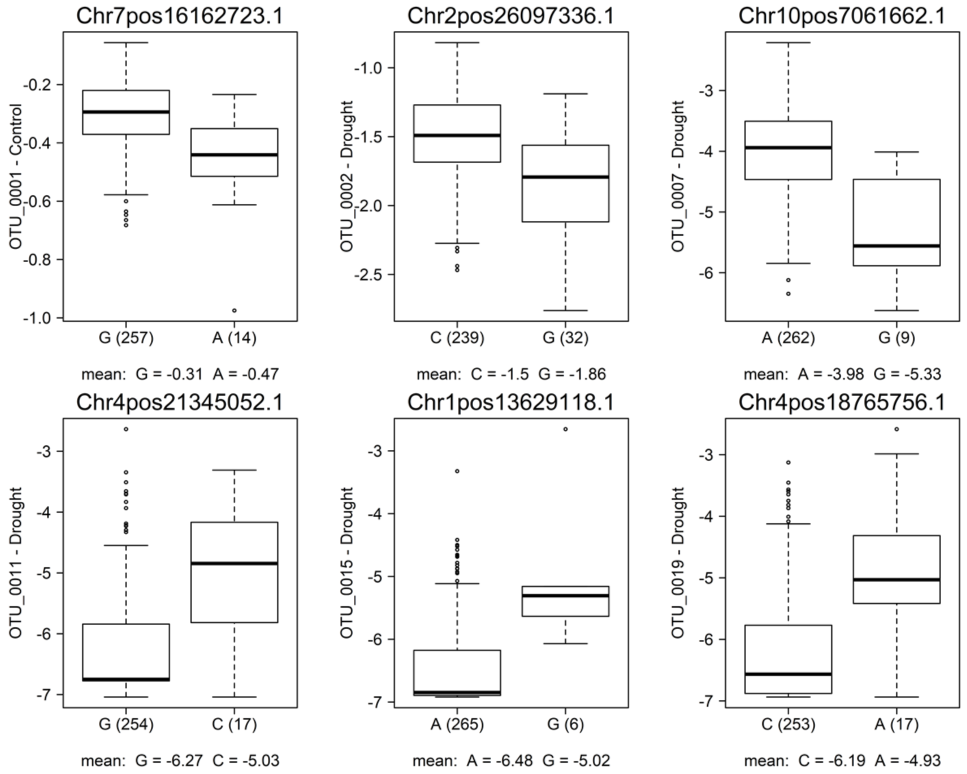

Fig. S4 Sequence-clusters contributing to plant yield. Spearman correlation analysis was performed with permuted *P* values (*P* values < 0.05) and RDA with yield as constrained axis. The top 18 sequence-clusters with the lowest *P* value and the higher yield contribution (RDA) are shown.

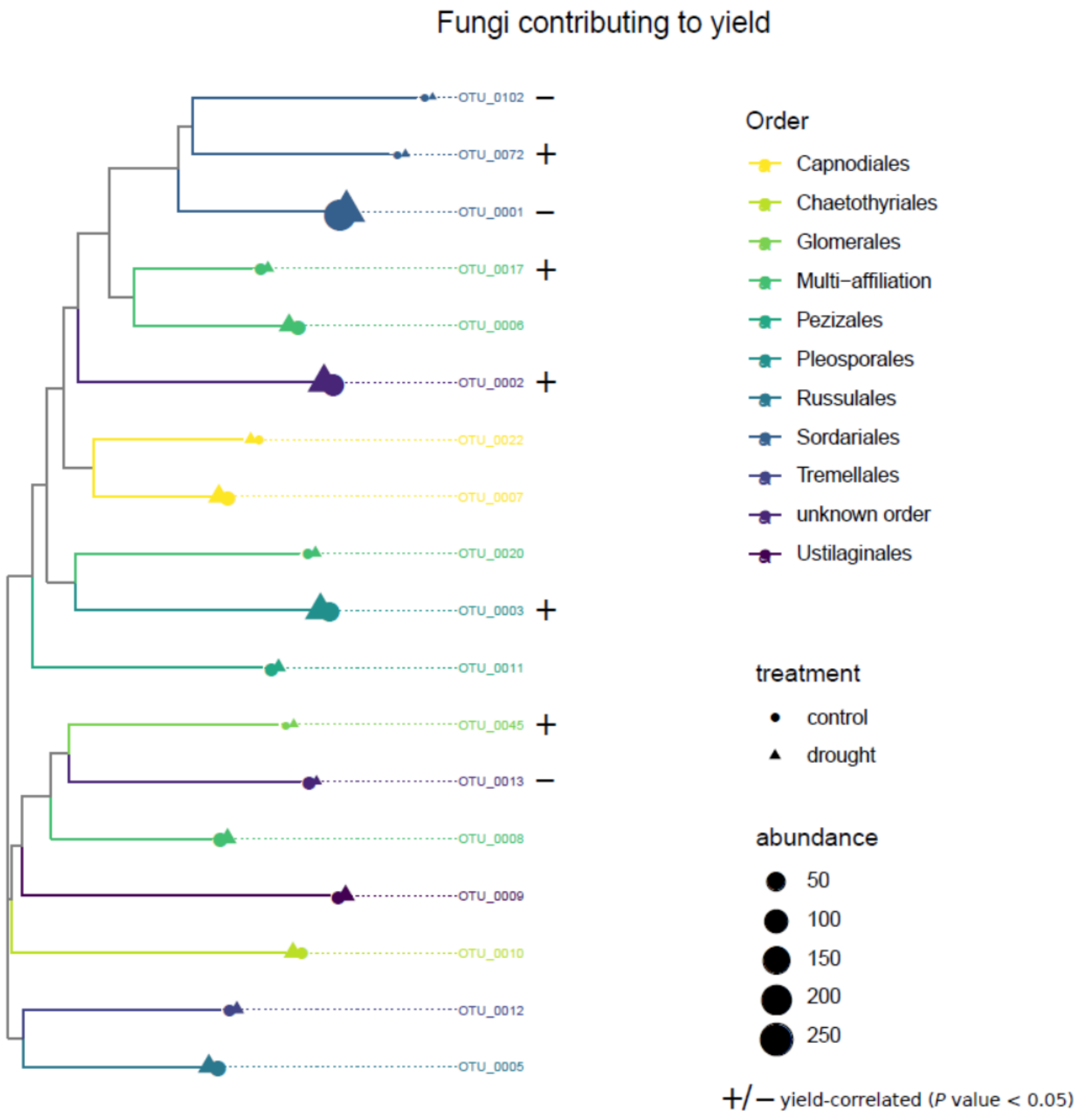

Table S1 List of SNPs found to be associated with specific fungal-clusters abundances and with drought-related fungal-clusters.

SNP	GWAS	OTU	loci number	gene functions
Chr1pos5498898.1	RDA-C	na	28	kinase/RALFL/keratin/MADS-box/plastocyanin/xylosyltransferase/AP2 protein/HD-Zip protein/MPPN
Chr1pos5530841.1	RDA-C	na	27	kinase/RALFL/Defensin/keratin/MADS-box/plastocyanin-like/xylosyltransferase
Chr2pos26097336.1	OTU-D	OTU_0002	25	kinase/aldehyde dehydrogenase/cytochrome/homeobox associated leucine zipper/hydrolase/protease/B-box zinc finger protein/glutaredoxin/polyamine oxidase
Chr4pos21345052.1	OTU-D	OTU_0011	23	kinase/cellulose synthase/calmodulin dependent kinase/chaperone GrpE/CorA-like magnesium transporter/dehydration-responsive element/F-box protein/Leucine-rich protein/nicotinate phosphoribosyltransferase/phospholipase/subtilisin
Chr2pos26151135.1	OTU-D	OTU_0002	22	kinase/aldehyde dehydrogenase/cytochrome/homeobox associated leucine zipper/hydrolase/protease
Chr10pos15125375.1	OTU-D	OTU_0019	19	BTB/POZ protein
Chr10pos15139435.1	OTU-D	OTU_0019	19	BTB/POZ protein
Chr4pos18765756.1	OTU-D	OTU_0019	13	RALFL/Defensin/CBS protein/EMB1688/exo70 exocyst complex/SWIB MDM2/vacuolar protein
Chr10pos7061662.1	OTU-D	OTU_0007	12	kinase/ligA
Chr4pos22658643.1	OTU-D	OTU_0019	5	transporter
Chr7pos16162723.1	OTU-C	OTU_0001	5	aminotransferase/chloroplast precursor/Fb34 fiber protein/HAD phosphatase/WRKY115
Chr4pos3632808.1	RDA-D	na	4	dioxygenase/peroxidase
Chr1pos13629118.1	OTU-D	OTU_0015	3	GATA zinc finger protein/importin/TPR repeat

Table S2 Sequence-clusters contributing to plant yield: detailed information.

		Control			*Drought*		
**OTU_ID**	**taxonomy**	**R†**	***P* value (permuted)***	**RDA scores****	**R†**	***P* value (permuted)***	**RDA scores****
OTU_0001	Chaetomiaceae fam.	-0.198	**0.001**	-5.88E-02	-0.007	0.904	-3.26E-03
OTU_0002	Ceratosphaeria spp.	0.159	**0.007**	1.64E-01	0.007	0.907	4.98E-03
OTU_0003	Paraconiothyrium spp.	0.276	**0**	**3.71E-01**	0.085	0.148	6.53E-02
OTU_0005	Russulales ord.	0.109	0.062	**1.62E-01**	0.012	0.841	**2.20E-02**
OTU_0006	Sordariomycetes class	0.051	0.381	**1.00E-01**	-0.03	0.615	**-6.58E-02**
OTU_0007	Cladosporium spp.	0.074	0.21	**1.35E-01**	0.066	0.264	**1.07E-01**
OTU_0008	Chytridiomycetes class	0.098	0.095	**1.68E-01**	0.01	0.87	**2.01E-02**
OTU_0009	Ustilaginales ord.	-0.099	0.09	**-1.92E-01**	-0.059	0.32	**-1.14E-01**
OTU_0010	Chaetothyriales ord.	0.048	0.409	**1.01E-01**	0.104	0.076	**2.30E-01**
OTU_0011	Boudiera spp.	0.121	0.039	**2.64E-01**	0.08	0.172	**1.66E-01**
OTU_0012	Tremellales ord.	-0.071	0.222	**-1.21E-01**	-0.046	0.43	**-7.84E-02**
OTU_0013	Peronosporomycetes phy.	-0.16	**0.006**	-2.44E-01	-0.038	0.513	-2.28E-02
OTU_0017	Apiosporaceae fam.	0.215	**0**	2.91E-01	0.024	0.676	2.47E-02
OTU_0020	Dothideomycetes class	0.076	0.194	**7.94E-02**	0.014	0.806	**1.74E-02**
OTU_0022	Mycosphaerellaceae fam.	0.108	0.065	7.67E-02	0.014	0.816	**1.79E-02**
OTU_0045	Glomerales ord.	0.144	**0.013**	5.80E-02	-0.054	0.36	-2.14E-02
OTU_0072	Chaetomium spp.	0.145	**0.014**	1.06E-02	na	na	na
OTU_0102	Sordariales ord.	-0.149	**0.01**	-7.35E-03	na	na	na

†represents Spearman correlation values between te OTU abundance and yield

*OTUs correlated with yield shown in bold (*P* value < 0.05)

**top 10 OTUs contributing to yield constrained axis in RDA analysis

Fig. S5 Mantel test plots. We studied the field position effect on the mycobiota results. Euclidean distances between samples (a) and correlogram (b) in control and drought samples.

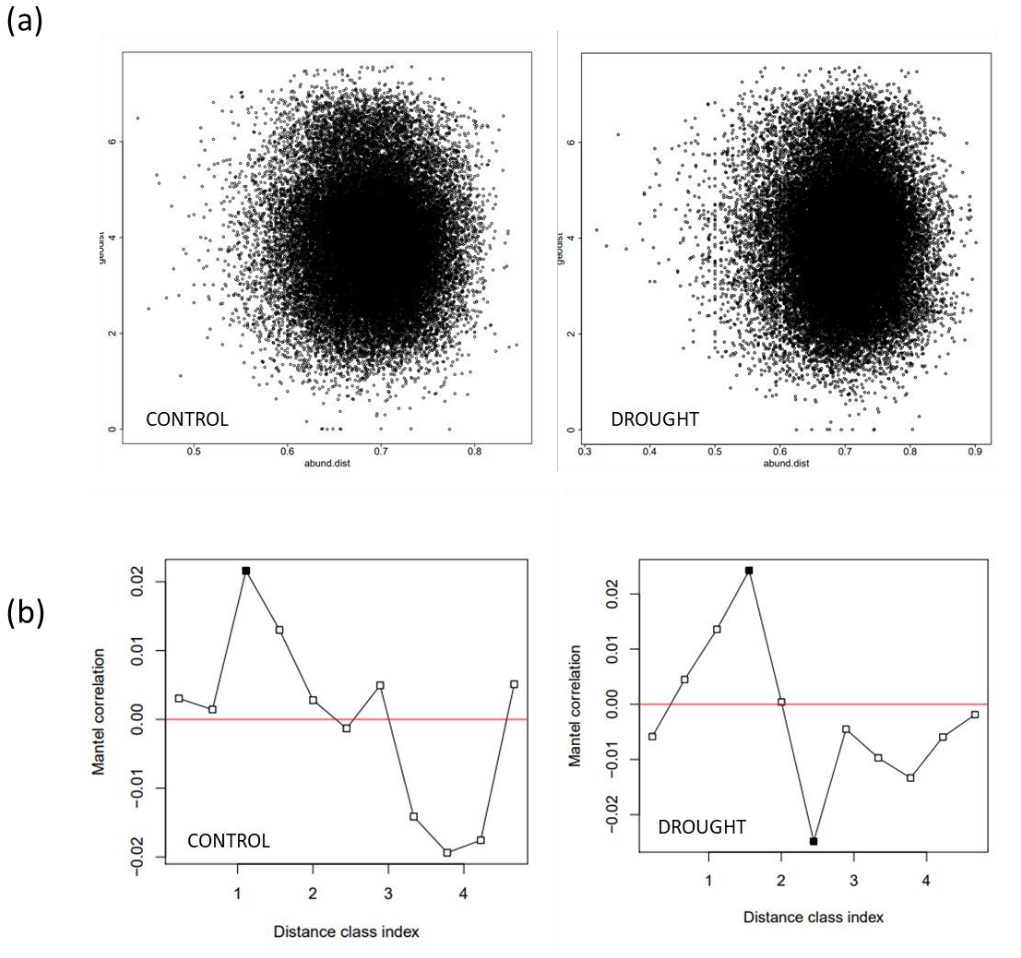

Fig. S6 Mycobiota found in the study. Bars represent total sum of abundances for each taxa. Phylum level is represented.

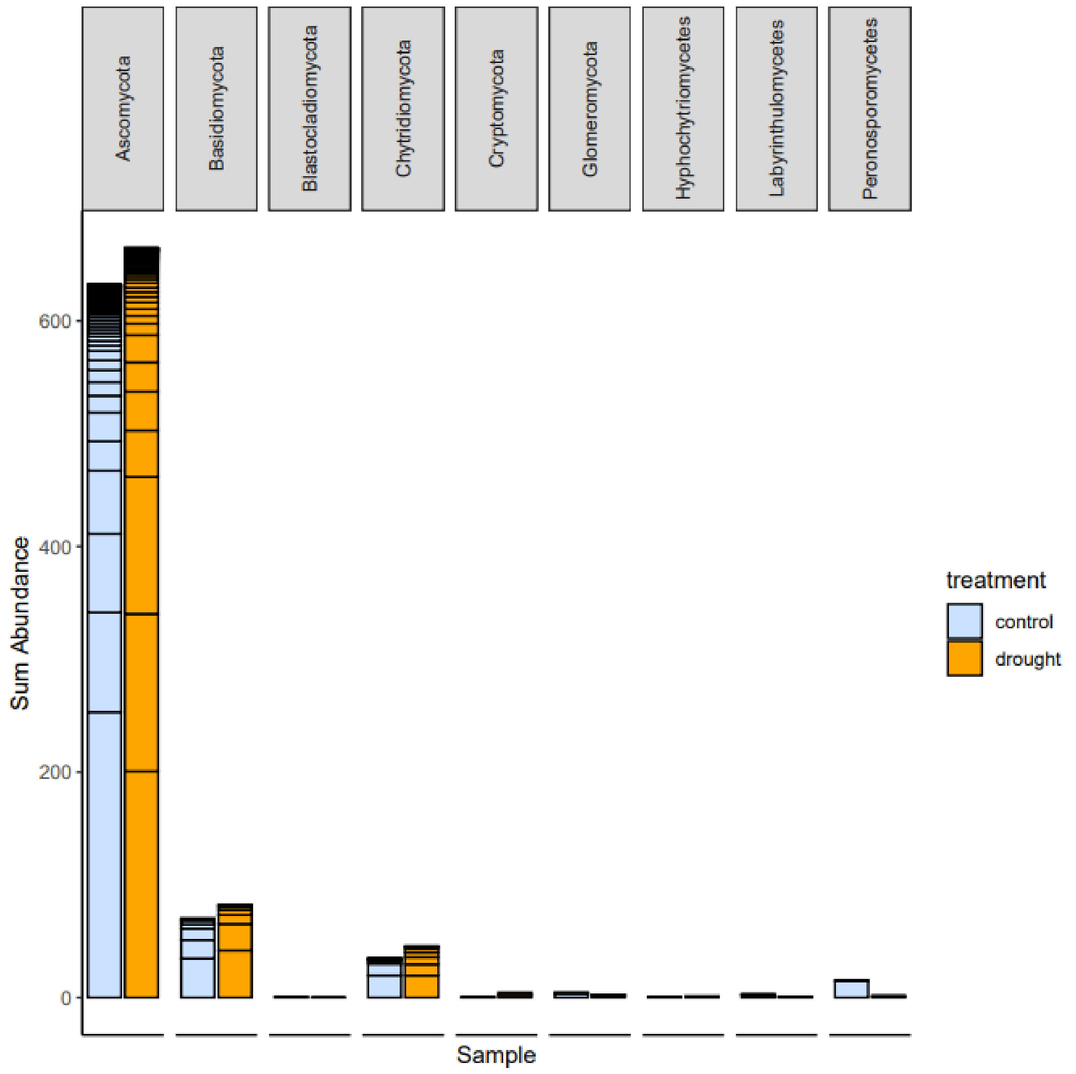

Supplement: Supplementary file 1 — Additional file 1. Supplementary Tables and Figures. Fig. S1: Fungal community description. Fig. S2: PCA of the mycobiota. Fig. S3: Boxplots with SNP allelic effect. Fig. S4: Sequence-clusters contributing to plant yield. Table S1: List of SNPs found to be associated with specific fungal-clusters abundances and with drought-related fungalclusters. Table S2: Sequence-clusters contributing to plant yield. Fig. S5: Mantel Test plots. Fig. S6: Mycobiota Phyla abundances. [file 12284_2023_641_MOESM1_ESM.pdf]
